# Divergent Root and Shoot Trait Strategies Shape Competition–Facilitation Shifts Between Conspecific and Heterospecific Neighbors Under Contrasting Soil Moisture Conditions

**DOI:** 10.1002/ece3.74586

**Published:** 2026-10-23

**Authors:** Jan Sher, Yaoqi Li, Yun‐Bing Zhang, Mengguang Han, Ghulam Mujtaba Shah, Jiao‐Lin Zhang

**Affiliations:** ^1^ Yunnan Key Laboratory of Forest Ecosystem Stability and Global Change, Xishuangbanna Tropical Botanical Garden Chinese Academy of Sciences Mengla Yunnan China; ^2^ Laboratory of Tropical Forest Ecology, Xishuangbanna Tropical Botanical Garden Chinese Academy of Sciences Mengla Yunnan China; ^3^ Department of Health and Environmental Sciences, School of Science Xi'an Jiaotong‐Liverpool University Suzhou China; ^4^ Ministry of Education Key Laboratory of Sustainable Forest Ecosystem Management, Northeast Asia Biodiversity Research Center, and School of Ecology Northeast Forestry University Harbin China; ^5^ Department of Botany Hazara University Mansehra Dhodial Pakistan

**Keywords:** biomass allocation, competition, functional traits, legumes, soil moisture

## Abstract

Soil moisture availability is a key driver of plant growth and competitive interactions among plant species. Trait differences among species are expected to influence competitive outcomes, with stronger competition often occurring among functionally similar species due to niche overlap. However, most studies have focused on aboveground traits, whereas belowground traits involved in water acquisition remain less explored. Here, we investigate how above‐ and belowground traits vary along soil moisture gradients and how they shape plant responses to intra‐ and interspecific competition. Seedlings of four tropical legume species were grown in a glasshouse for 30 weeks under three soil moisture levels (low, medium, and high) and three neighbor treatments: intraspecific competition, interspecific competition, and no‐competition. At harvest, above‐ and belowground morphological and physiological traits, as well as biomass accumulation, were measured using standardized protocols. Plant performance was strongly influenced by competition and soil moisture. Intraspecific competition increased belowground traits, including specific root length, average root diameter, and root–shoot ratio, particularly under low moisture conditions, and resulted in greater belowground biomass. In contrast, interspecific competition enhanced aboveground traits, including leaf area, average leaf mass, and specific leaf area, leading to higher aboveground biomass and total biomass. Nodule number and nodule dry mass increased with soil moisture, whereas antioxidant enzyme activity was highest under low moisture. Relative interaction index analyses further showed stronger belowground competitive effects under intraspecific interactions and greater aboveground facilitative effects under interspecific interactions. These findings highlight that plant–plant interactions in tropical legumes are highly context‐dependent, with neighbor identity and soil moisture together influencing competitive outcomes. Divergent below‐ and aboveground investment strategies reveal how plants adjust resource allocation, shaping growth performance under changing environmental conditions.

## Graphical abstract

Soil moisture availability strongly influences plant–plant interactions, yet the role of coordinated root and shoot traits in shaping competition and facilitation remains poorly understood. Using a 7‐month glasshouse experiment with four tropical legume species, we demonstrate that soil moisture and neighbor identity jointly regulate plant strategies, with intraspecific interactions enhancing belowground investment and interspecific interactions promoting aboveground growth and facilitative effects. Our findings reveal that root–shoot trait coordination is a key mechanism driving shifts between competition and facilitation under changing moisture conditions.

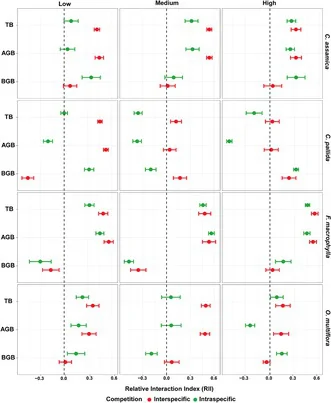

## 1 Introduction

Soil moisture is one of the key environmental factors regulating plant carbon assimilation, water and nutrient uptake, growth and survival, particularly in terrestrial ecosystems under climate change (Asefa et al. 2022; Berg and Sheffield 2018; Wang et al. 2019; Zhang et al. 2026; Zhang and Tielbörger 2019). Increasing drought and altered precipitation regimes are expected to modify soil water availability, thereby generating strong spatial and temporal variation in soil moisture. Such variation can strongly affect plant performance, species interactions and community dynamics (Zhang et al. 2026). Plants respond to contrasting water environments by adjusting functional traits and resource‐use strategies, including traits associated with leaf carbon gain, root water acquisition and whole‐plant biomass allocation, which shape their physiological performance and competitive outcomes (Faucon et al. 2017; Ghosh et al. 2018; Liu et al. 2023; Van Dyke et al. 2022). Understanding how plant functional traits respond to soil moisture gradients and how these responses are modified by plant competition is therefore critical for predicting vegetation dynamics, community structure, and ecosystem functioning under ongoing environmental changes (Gratani 2014; Heilmeier 2019; Rao et al. 2024).

Trait‐based ecology has traditionally focused on aboveground organs, particularly leaves and shoots, because they are closely linked to light capture, carbon assimilation, and growth rate (Lorts and Lasky 2020; Anderegg et al. 2021). By contrast, belowground traits remain less well understood, despite their central role in water and nutrient acquisition and whole‐plant performance (Bardgett et al. 2014; Laliberté 2017; Asefa et al. 2022). Recent studies show that root traits provide important information about plant ecological strategies that cannot be explained by aboveground traits alone, highlighting the need to consider both above‐ and belowground traits jointly when studying plant adaptation and resource use (Carmona et al. 2021). Leaf economics spectrum describes a fundamental continuum from rapid resource acquisition to conservative resource use in leaves, providing a major framework for predicting plant ecological strategies (Wright et al. 2004; Májeková et al. 2021). Whether comparable economic trade‐offs extend consistently belowground, however, remains unresolved. Recent work suggests that root traits are organized within a multidimensional root economics space, structured by both a resource acquisition–conservation axis and a mycorrhizal collaboration axis (Bergmann et al. 2020). Whole‐plant economic theory therefore predicts that acquisitive leaf traits, such as high specific leaf area and leaf nitrogen concentration, may be coordinated with acquisitive root traits, such as high specific root length and root nitrogen concentration. However, empirical evidence remains mixed: some studies report positive leaf–root trait associations, particularly under resource limitation, whereas others find weak or context‐dependent relationships, suggesting that above‐ and belowground traits may respond independently to environmental variation and species interactions (Brunner et al. 2015; Weemstra et al. 2016; Weigelt et al. 2021).

The functional equilibrium hypothesis provides a complementary framework by explaining how plants adjust biomass allocation in response to resource limitations (Brouwer 1963; Poorter and Nagel 2000). According to this hypothesis, plants should allocate more biomass to roots when belowground resources such as water or nutrients are limiting, and more biomass to shoots and leaves when light is limiting. Along with soil moisture gradients, drought may therefore increase belowground investment, while high soil moisture may promote aboveground growth (Asefa et al. 2022). Some species adopt conservative strategies by producing thicker, deeper, and longer‐lived roots that enhance persistence under water limitation (Larson and Funk 2016; Cheng et al. 2025). Others develop finer roots with high specific root lengths and high surface area to improve water uptake from the soil (Wasson et al. 2012; Comas et al. 2013; Cheng et al. 2025). Under wetter conditions, plants may reduce belowground allocation with thinner and short‐lived roots and invest more in leaves and shoots, although responses can vary among species and competitive environments (Larson and Funk 2016; Foxx and Fort 2019). These contrasting strategies suggest that soil moisture gradients may alter not only total biomass allocation but also the coordination between leaf and root traits.

Plant responses to soil moisture are further shaped by biotic stressors. In natural communities, plants grow with both conspecific and heterospecific neighbors, which can strongly influence resource availability, trait expression, and biomass allocation (Fitzpatrick et al. 2019; Foxx and Fort 2019; Asefa et al. 2022). Conspecific neighbors are often expected to impose strong competition because they share similar resource requirements and occupy similar niches. Heterospecific neighbors, by contrast, may compete less strongly if they differ in rooting depth, resource acquisition strategies, or temporal patterns of resource use. The stress gradient hypothesis (SGH) proposes that the balance between competition and facilitation among plants changes along environmental stress gradients, with facilitation becoming more important under stressful conditions (Callaway and Mahall 2007; Maestre et al. 2009; Holmgren and Scheffer 2010). However, this pattern is not universal and depends on the stress type and severity (Armas et al. 2011). Under drought, facilitation may occur at moderate stress levels but weaken or disappear under severe stress when plants compete strongly for limited water (Maestre et al. 2009). Thus, the outcomes of plant interactions along soil moisture gradients are likely to depend on both water availability and neighbor identity.

Tropical ecosystems provide an important but underrepresented context for testing these ideas. Many tropical regions experience strong seasonal variation in rainfall, which can generate strong temporal fluctuations in soil moisture. Legumes are particularly relevant in these ecosystems because their nitrogen‐fixing capacity, diverse root strategies, and strong effects on soil fertility can strongly influence soil fertility and nutrient cycling (Thomas 1992; Kebede 2021; Rajput et al. 2025). The Xishuangbanna region of southwestern China has a tropical monsoon climate, with more than 80% of annual precipitation occurring during the wet season (May–October), whereas the dry season (November–April) is characterized by low rainfall and frequent fog (Asefa et al. 2022; Cao et al. 2006; Liu et al. 2014). This pronounced seasonal contrast creates large fluctuations in soil moisture, making water availability a major driver of plant growth and plant–plant interactions in this region. Yet most studies examining competition along soil moisture gradients have focused on temperate ecosystems, leaving the trait‐based mechanisms underlying tropical plant interactions comparatively poorly understood.

Here, we conducted a controlled experiment with four tropical legume species grown either alone, with conspecific neighbors (intraspecific competition), or with heterospecific neighbors (interspecific competition) across three soil moisture treatments: low, medium, and high. The medium treatment was designed to represent typical growing conditions, whereas the low and high treatments simulated reduced and increased water availability. This experimental design allowed us to examine how soil moisture and neighbor identity jointly influence plant functional traits, biomass allocation, interaction outcomes, and relative performance in tropical ecosystems. Specifically, we addressed two questions: (I) How do above‐ and belowground resource‐acquisition traits respond to soil moisture gradients and neighbor identity? We hypothesized that low soil moisture and neighbor identity would regulate plant resource acquisition strategies. Under low soil moisture, plants would enhance belowground resource acquisition through greater expression of root traits, particularly under intraspecific competition, whereas higher soil moisture and interspecific interactions would favor shifts toward aboveground resource acquisition strategies (H1). (II) How do soil moisture gradients and neighbor identity influence plant interactions and biomass performance? We hypothesized that neighbor identity would regulate the balance between competition and facilitation across soil moisture gradients. Conspecific interactions were expected to impose stronger competitive effects due to shared resource demands, leading to greater biomass reduction. In contrast, heterospecific interactions were expected to reduce competitive intensity or promote facilitation through differences in resource acquisition strategies, resulting in higher biomass production (HII). Together, these hypotheses provide a framework for understanding how resource acquisition strategies and plant interactions shape biomass responses of tropical legumes under variable water availability.

## 2 Materials and Methods

### 2.1 Seed Collection and Germination

Four tropical legume species from the family Fabaceae were selected for this study: *Crotalaria pallida* var. *obovata* (G. Don) Polhill, *Crotalaria assamica* Benth., *Flemingia macrophylla* (Willd.) Kuntze ex Merr., and *Ototropis multiflora* (DC.) H.Ohashi & K.Ohashi. Legumes were selected because they are ecologically important and functionally diverse components of tropical ecosystems. Through symbiotic associations with nitrogen‐fixing rhizobia, legumes can contribute substantially to nutrient cycling, soil fertility, and ecosystem productivity (Saikia et al. 2020). Their growth and performance are also closely linked to belowground resource acquisition and are often sensitive to variation in water availability. These characteristics make legumes an ideal study system for examining how soil moisture gradients influence functional trait responses and plant–plant interactions.

All four species are native to China and naturally distributed in the tropical forests of Xishuangbanna, Yunnan Province, Southwest China. We collected approximately 300–400 seeds of each species from multiple maternal plants in natural populations to minimize potential maternal effects. Seed were stored in the seed bank of the Xishuangbanna Tropical Botanical Garden, Chinese Academy of Sciences (21°54′30″ N, 101°46′59″ E; 580 m a.s.l.). Seed viability was assessed before sowing, and all species presented high germination rates (> 80%). Because the four species differed in germination timing, seeds of the slower‐germinating species, *F. macrophylla* and *O. multiflora* , were sown earlier than those of the faster‐germinating species, *C. pallida* and *C. assamica* (Sher et al. 2026). This staggered sowing ensured that the seedlings were at comparable developmental stages at transplantation.

Before sowing, seeds were soaked in water overnight to promote germination and then placed in Petri dishes in a controlled growth chamber. After approximately 4 weeks, when the seedlings had developed three to five true leaves, healthy and uniformly sized individuals were selected and transplanted into pots in November 2023. Some seedlings died during the first month after transplanting. Dead seedlings were replaced with healthy seedlings from the extra seedlings prepared before the experiment. No replacement was conducted after the first month, and the remaining plants were maintained until harvest. The experiment was conducted in a glasshouse at Xishuangbanna Tropical Botanical Garden. The region has a tropical monsoon climate, with a mean annual temperature of approximately 21.8°C and annual precipitation of approximately 1493 mm (Asefa et al. 2022; Cao et al. 2006; Liu et al. 2014). During the experiment, glasshouse temperature was maintained at approximately 28°C–31°C during the 12‐h daytime period and 20°C–24°C during the 12‐h nighttime period, with relative humidity ranging between 60% and 80%. These conditions fall within the range commonly experienced during the warm growth season in the species' native habitats. All plants were grown under the same glasshouse light conditions throughout the experiment. The experimental soil was collected from a nearby field and was characterized as sandy soil with low water‐holding capacity, consistent with the soil conditions reported for the natural habitats of the studied species. Before pot filling, the soil was sieved and thoroughly homogenized to minimize variation in soil properties among pots and ensure comparable initial conditions across treatments.

### 2.2 Experimental Design and Treatments

A fully factorial pot experiment was conducted to examine the effects of soil moisture availability and neighbor identity on plant functional traits, biomass allocation and plant–plant interaction outcomes. The experiment included three soil moisture levels and three neighbor treatments. Soil moisture treatments consisted of low (dry), medium (control), and high (wet) water availability, corresponding to target volumetric water content (VWC) of 10%, 20%, and 35%, respectively. These levels were selected to represent the natural range of soil moisture observed in Xishuangbanna tropical forests between the dry and wet seasons. For example, soil moisture at nearby field sites can range from approximately 10% during the dry season to 30%–40% during the rainy season (Li et al. 2012; Asefa et al. 2022). Soil moisture was maintained by daily watering. Soil VWC was monitored using a dielectric conductivity–based soil moisture probe connected to an EM50 data logger (METER Group Inc., Pullman, WA, USA). Water was added to maintain the target VWC of 10%, 20%, and 35%. The amount of water added was determined based on VWC measurements from randomly selected pots (20–30 pots across the experiment). The same pots were repeatedly monitored during the experiment to guide watering and ensure stable moisture conditions. After watering, VWC was measured again in the monitored pots to confirm that the target moisture levels were achieved and maintained throughout the experiment (Figure S1). All pots were free draining, allowing excess water to drain after irrigation and preventing prolonged water accumulation. The 35% VWC treatment was considered a high soil moisture condition rather than a waterlogged treatment, although higher moisture levels may have reduced soil aeration compared with the lower moisture treatments.

Neighbor treatments included no‐neighbor control, in which a single focal plant was grown alone; intraspecific competition, in which individuals were grown with conspecific neighbors (paired seedlings belonged to the same species); and interspecific competition, in which individuals were grown with heterospecific neighbors (seedlings as two different species). Plants were grown in plastic pots (22 cm in height and 25 cm in diameter). Each pot was filled with approximately 10 kg of homogenized sandy soil (> 70% sand content), providing sufficient rooting volume to support plant growth across competition treatments and to maintain stable soil moisture conditions under repeated watering. Pots were randomly arranged within the glasshouse and repositioned regularly to reduce potential effects of spatial heterogeneity in light and temperature. In the interaction treatments, four seedlings were planted per pot at equal spacing, with neighboring individuals placed approximately 6 cm apart to allow both above‐ and belowground interactions. Plant density was therefore kept constant between the intraspecific and interspecific treatments, ensuring that differences between these interaction types reflected neighbor identity rather than variation in plant density.

For the intraspecific competition treatment, each pot contained four individuals of the same species. Each species × soil moisture combination was replicated three times, resulting in 36 pots. For the interspecific treatment, all six pairwise combinations of the four species were used. Each species combination was grown under each of the three soil moisture treatments, with three replicate pots per species pair and soil moisture level. Each pot contained four individuals, with two individuals from each species, resulting in 54 pots. For the no‐competition (control) treatment, each species was grown individually under each soil moisture level, with five replicate pots per species and moisture treatment, resulting in 60 pots. In total, the experiment included 150 pots and 420 seedlings.

### 2.3 Plant Harvest, Biomass and Functional Trait Measurements

The plants were harvested after 30 weeks of growth (June 2024). At harvest, all individuals within each pot were separated by species and measured individually. Fresh plant material was used to measure stem length (cm; main stem length) and total leaf area (cm^2^) (Tomlinson et al. 2012; Sher et al. 2023, 2024; Sher, Zhang, and Zhang 2025). After fresh measurements, leaves, stems, and roots were dried at 65°C for 48 h to constant mass. The dry masses of the roots, stems, and leaves were then recorded separately. These measurements were used to calculate the total biomass (TB, unit as g) as the sum of leaf, stem, and root dry mass, aboveground biomass (AGB, g) as the sum of leaf and stem dry mass, and belowground biomass (BGB, g) as total root dry mass. Plant biomass is used as a reliable indicator of plant performance and fitness (Younginger et al. 2017; Sher et al. 2024; Sher, Zhang, and Zhang 2025). For each plant, the number of root nodules was counted, and nodules were dried and weighed to estimate nodule dry mass (NDM) as an indicator of investment of legume plants in symbiotic nitrogen fixation.

To further evaluate plant performance, we measured key above‐ and belowground traits associated with resource acquisition and biomass allocation. Fully expanded leaves were scanned at 300 dpi via an Epson V850 Pro scanner. Leaf area (LA) was quantified from scanned images, and specific leaf area (SLA; cm^2^ g^−1^) was then calculated as leaf area divided by leaf dry mass via R software. Average leaf mass (ALM; g) was calculated as total leaf dry mass divided by leaf number. Specific stem length (SSL; m g^−1^) was calculated as stem length divided by stem dry mass. For the root traits, fine roots with diameters smaller than 2 mm were separated, cleaned, and scanned at 600 dpi with the same scanner. Root images were analyzed via WinRHIZO (Rhizo Explorer version) to determine specific root length (SRL; m g^−1^ root mass) and average root diameter (ARD; mm). SRL was calculated as total fine‐root length divided by fine‐root dry mass. These traits were selected because they reflect variation in plant strategies for acquiring key resources such as light, nutrients, and water, and because they are commonly used to characterize above‐ and belowground resource‐use strategies (Tomlinson et al. 2012, 2019; Perez‐Harguindeguy et al. 2016; Sher, Zhang, and Zhang 2025).

### 2.4 Antioxidant Enzyme Activity and Oxidative Stress Indicators

Antioxidant enzyme activities were measured to examine physiological responses of plants to soil moisture and neighbor treatments. Variation in soil water availability and plant competition can alter the production of reactive oxygen species, potentially leading to oxidative stress and cellular damage. Antioxidant enzymes play a key role in protecting plant tissues by detoxifying harmful oxidative compounds, thereby helping plants maintain physiological function and stress tolerance under both drought and well‐watered conditions. We followed standard protocols adopted by previous studies (Jungklang et al. 2017; Yu and Rengel 1999; Zhang and Kirkham 1994). To obtain a representative sample for each plant, leaves were collected from every individual in each replicate across all treatments. Three leaves from different positions of each plant (upper, middle, and lower parts) were combined to create one composite sample. Approximately 0.1 g of fresh leaf tissue from each composite sample was used for enzyme activity measurements. The samples were wrapped in aluminum foil, rapidly frozen in liquid nitrogen, and stored at ultra‐low temperature until analysis to preserve enzyme activity. Before enzyme extraction, the frozen leaf samples were ground into a fine powder using a precooled mortar and pestle, and the powder was mixed with 1 mL of the corresponding extraction buffer for enzyme analysis. The homogenized samples were centrifuged at 12,000 rpm for 15 min at 4°C, and the clear supernatant was used for enzyme assays. The activities of catalase (CAT), superoxide dismutase (SOD), peroxidase (POD), and malondialdehyde (MDA, an indicator of lipid damage) were determined via commercial assay kits (Beijing Solarbio Science & Technology Co. Ltd., China) following the manufacturer's instructions. Enzyme activity calculations followed standard methods described in previous studies (Jungklang et al. 2017; Deng et al. 2018; Wang and Wei 2022).

### 2.5 Statistical Analysis

We applied linear mixed‐effects models (LMMs) to test the effects of soil moisture, competition, and their interaction on plant biomass, functional traits, nodulation traits, and physiological variables. Models were fitted using the *lme4* package (Bates et al. 2015). Soil moisture and competition treatment, including their interaction, were treated as fixed effects, while PotID and species identity were included as random effects to account for shared pot effects and species‐level variation, respectively (Sher et al. 2024; Sher, Zhang, and Zhang 2025). Each response variable, such as biomass, resource acquisition traits, and antioxidant enzyme activities, was analyzed separately. Response variables were inspected for normality and homogeneity of variance using residual diagnostics. Variables were log root‐transformed when necessary to improve model assumption. The significance of the model coefficients was assessed using *t*‐tests with Satterthwaite's approximation for degrees of freedom, and model fit was evaluated via marginal and conditional *R* ^2^ values calculated with the *r.squaredGLMM* function in the *MuMIn* package (Nakagawa and Schielzeth 2013). Subsequently, pairwise group comparisons were conducted using Tukey's HSD test using the glht function in the multcomp package.

To evaluate how trait–performance relationships differed among neighbor environments, we examined correlations between biomass variables and resource‐acquisition traits separately for no‐competition, intraspecific, and interspecific treatments. These analyses were used to determine whether the relationships between functional traits and biomass accumulation differed under contrasting competitive environments. We further conducted principal component analysis (PCA) to examine multivariate patterns of trait coordination across soil moisture and neighbor treatments. PCA was performed on the individual × trait matrix using the prcomp function in R (Dixon 2003; Oksanen 2013). Prior to PCA, trait distributions were examined, and variables showing strong skewness were log‐transformed to improve normality. The transformed traits included ARD, SRL, RSR, SSL, ALM, LA, SLA, BGB, AGB, and SOD, whereas traits that met distributional assumptions were retained without transformation. All trait variables were standardized by mean‐centering and scaling to unit variance before PCA to account for differences in measurement units among traits.

Fifteen functional traits representing belowground resource acquisition, aboveground resource acquisition, biomass accumulation, and physiological responses were retained for the final PCA. TB was excluded to avoid redundancy with AGB and BGB components. The first five principal components were examined to characterize major axes of trait variation, while the first two principal components were visualized to illustrate overall trait coordination patterns associated with soil moisture and neighbor competition. Trait contributions to each principal component were calculated from normalized squared loadings, with contribution values expressed as percentages and summed to 100% within each principal component. PCA loadings and trait–principal component correlation coefficients were used to identify traits contributing most strongly to multivariate trait variation. All statistical analyses were performed using R version 4.2.2 (R Core Team 2022).

#### 2.5.1 Relative Biomass Change

To quantify the effect of neighbor presence on plant growth performance, we calculated the relative change in individual plant total biomass (%) across different soil moisture conditions. For each species and soil moisture treatment, the mean individual total biomass of plants grown without neighbors was used as the baseline reference. Relative biomass change under intraspecific and interspecific neighbor treatments was calculated according to the following equation:

(1) $$\text{Relative Change}\;\left(\%\right)=\frac{\;{\mathrm{TB}}_{\text{comp}}-{\mathrm{TB}}_{\text{control}}\;}{{\mathrm{TB}}_{\text{control}}}\times 100$$

where $TB_{\text{comp}}$ represents the mean individual total biomass (g plant^−1^) of a species grown under intraspecific or interspecific neighbor treatments, and $TB_{\text{control}}$ represents the mean individual total biomass (g plant^−1^) of the same species grown without neighbor under the corresponding soil moisture treatment. Positive values indicate increased individual biomass in the presence of neighbors, whereas negative values indicate reduced individual biomass relative to the no‐competition treatment. This approach allowed us to evaluate the magnitude and direction of neighbor effects on plant biomass production among species and soil moisture conditions.

#### 2.5.2 Relative Interaction Index (RII)

The relative interaction index (RII) was calculated to quantify the strength and direction of plant–plant interactions following (Armas et al. 2004) and subsequent applications (e.g., Carmona et al. 2019; Donnelly et al. 2023). RII was calculated for biomass variables, including BGB, AGB, and TB, across soil moisture treatments. Because all plants were harvested and measured individually, RII calculations were based on individual plant biomass (g plant^−1^). For each species and soil moisture condition, the mean individual biomass of plants grown without neighbors was used as the species‐specific control reference. The RII for plants grown under intraspecific and interspecific neighbor treatments was calculated as:

(2) $$\mathrm{RII}=\frac{B_{\text{Comp}}-B_{\text{Control}}}{B_{\text{Comp}}+B_{\text{Control}}}$$

where $B_{\text{Comp}}$ represents the biomass of an individual plant grown under intraspecific or interspecific neighbor treatments, and $B_{\text{Control}}$ represents the mean individual biomass of the same species grown without neighbor under the corresponding soil moisture condition. RII values range from −1 to +1, with negative values indicating reduced biomass relative to the no‐neighbor control (competitive effects), positive values indicating increased biomass relative to the no‐neighbor control (facilitative effects), and values close to zero indicating neutral interactions.

#### 2.5.3 Relative Height Growth Rate (RHGR)

The relative height growth rate (RHGR) was calculated to assess the growth performance of each species under different competition and soil moisture treatments. Plant height was measured at transplantation (T_0_) and again at final harvest (T_1_). The RHGR was calculated via the natural logarithm (ln) to standardize growth relative to initial plant size, which allows meaningful comparisons among individuals of different starting heights. The RGR was then calculated via the following formula:

(3) $$\text{RHGR}=\frac{\ln\left(H_{1}\;\right)-\ln\left(H_{0}\;\right)}{\Delta t}$$

where $H_{0}$ and $H_{1}$ represent the initial and final plant heights, respectively, and Δt represents the duration of the experiment (7 months). This metric quantifies the proportional increase in plant height per unit time while accounting for differences in initial plant size (Yang et al. 2025).

## 3 Results

### 3.1 Responses of Below‐ and Aboveground Resource‐Acquisition and Allocation Traits to Competition and Soil Moisture

Below‐ and aboveground resource‐acquisition traits showed significant variation among competition treatments and soil moisture levels (Table 1; Figure 1a,b). Belowground acquisition traits, including ARD and SRL, were strongly influenced by the interaction between competition and soil moisture. ARD increased under water‐limited conditions, with plants grown with intraspecific neighbors showing greater ARD than those grown with interspecific neighbors or without competition. This pattern was strongest under low soil moisture and gradually decreased under medium and high soil moisture levels. Plants under intraspecific competition generally showed higher SRL than those under interspecific competition and no‐competition across soil moisture levels, although the magnitude of these differences varied among moisture treatments. RSR showed an interactive response to competition and soil moisture (Table 1; Figure 1c). Under low soil moisture, plants grown under intraspecific competition had higher RSR than those grown under interspecific competition and no‐competition, whereas interspecific competition had no significant effect on RSR across soil moisture levels. Nodule traits were also strongly influenced by the interaction between competition and soil moisture (Table 1; Figure 1d). Nodule number (NN) increased with increasing soil moisture, with plants grown with intraspecific neighbors producing more nodules under high soil moisture. Similarly, nodule dry mass (NDM) increased with soil moisture availability, with greater NDM under high soil moisture and lower NDM under low soil moisture. In contrast, competition had relatively limited effects on NDM compared with the stronger effect of soil moisture availability (Table 1; Figure 1e).

**TABLE 1:** Mixed‐effects model results for above‐ and belowground resource‐acquisition traits.

	ARD (log)	SRL (log)	RSR (log)	NN	NDM	SSL (log)	ALM (log)	LA (log)	SLA (log)
Random effects									
PotID	0.06	0.43	0.57	1.72	7.99	0.45	0.35	0.15	0.30
Species	0.03	0.31	0.24	0.21	0.00	0.26	0.36	0.14	0.17
Residual	0.14	0.96	1.02	4.48	13.93	0.72	0.73	0.28	0.76
Fixed effects									
Intercept	**−0.873**	**0.764**	**−2.233**	**15.90**	**105.86**	**0.424**	**−4.685**	**3.087**	**3.705**
Competition (C): Inter	−0.124	−0.290	−0.557	**5.586**	7.561	**−1.109**	**1.131**	**0.240**	−0.146
Competition (C): Intra	0.138	**0.822**	**−0.855**	**−0.962**	2.773	−0.408	**0.756**	−0.172	**−0.451**
Soil moisture (SM): High	**−0.431**	0.481	**−1.135**	**3.000**	**9.300**	**−0.764**	**0.921**	**0.170**	0.219
Soil moisture (SM): Low	−0.014	0.039	0.024	**−4.500**	**−15.43**	−0.052	0.119	**−0.543**	−0.085
C: Inter × SM: High	**0.443**	−0.502	0.804	−2.097	−1.215	**0.610**	**−0.763**	0.003	0.237
C: Intra × SM: High	**0.262**	**−0.812**	**1.186**	**9.645**	2.233	**0.375**	**−0.720**	0.076	0.476
C: Inter × SM: Low	**0.295**	−0.156	0.312	**−4.597**	**−11.88**	0.440	−0.255	0.010	**−0.829**
C: Intra × SM: Low	0.222	**−0.879**	**1.303**	**6.520**	−5.043	**0.771**	−0.119	0.309	0.229
Explanatory power									
*R* ^2^m	0.244	0.123	0.132	0.514	0.4300	0.161	0.122	0.435	0.233
*R* ^2^c	0.363	0.325	0.369	0.578	0.571	0.451	0.419	0.626	0.415

*Note:* Species and PotID were included as random effects, whereas competition treatments and soil moisture gradient were included as fixed effects. Competition was treated as a categorical factor with no competition as the reference level, and medium soil moisture was used as the reference category for the soil moisture gradient. Transformations applied to satisfy normality assumptions are indicated. Standard deviations of random effects and reference categories for fixed effects are reported. Coefficients were evaluated using *t*‐tests with Satterthwaite‐adjusted degrees of freedom, and significant effects (*p* < 0.05) are shown in bold.

**FIGURE 1:**
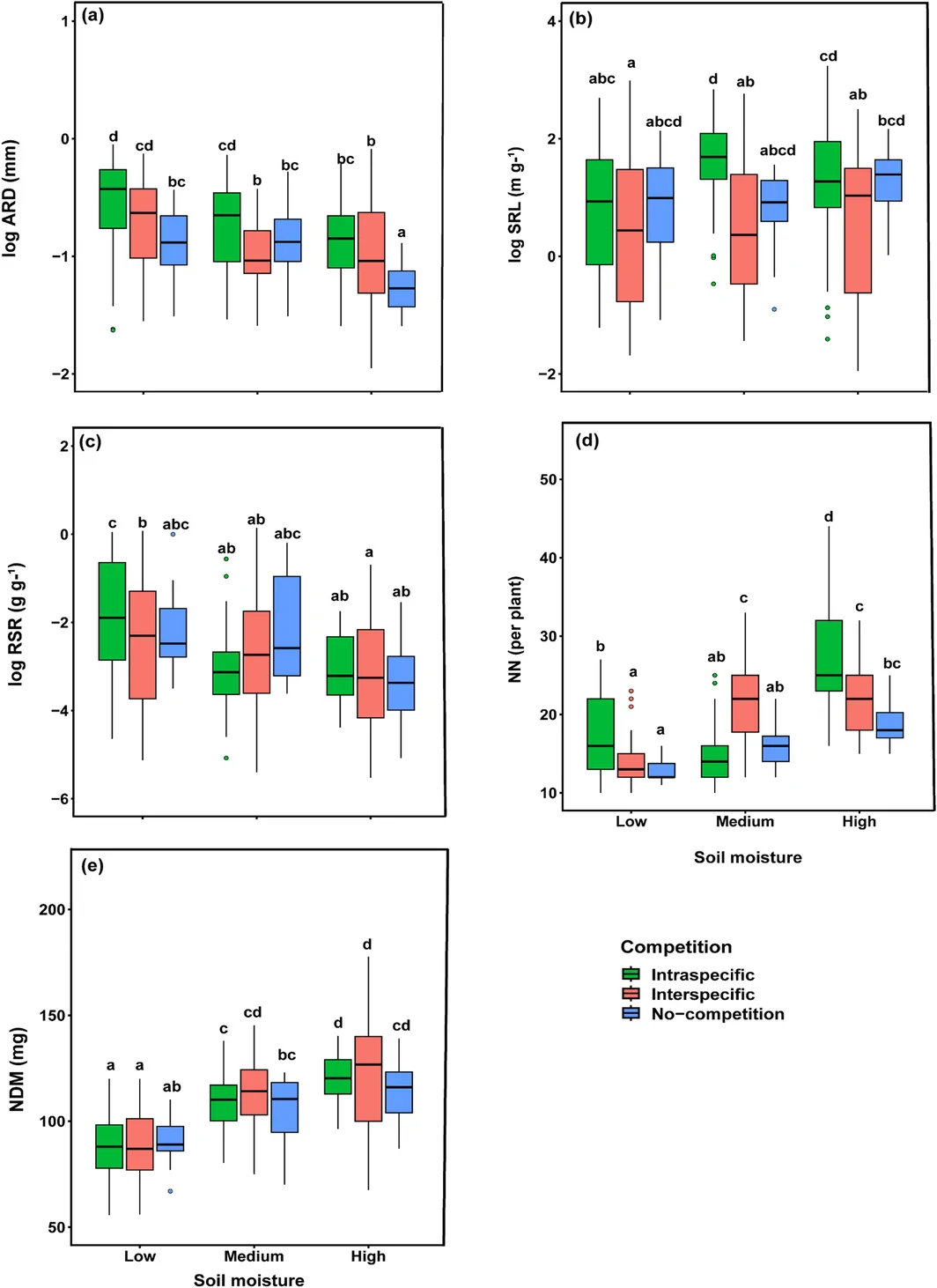
Effects of interspecific, intraspecific, and no‐competition treatments on belowground acquisition, biomass allocation, and symbiotic nitrogen acquisition traits across low, medium, and high soil moisture levels. Measured traits include (a) average root diameter (ARD, mm) and (b) specific root length (SRL, m g^−1^) as belowground acquisition traits; (c) root‐to‐shoot ratio (RSR, g g^−1^) as a biomass allocation trait; and (d) nodule number (NN, per plant) and (e) nodule dry mass (NDM, mg) as symbiotic nitrogen acquisition traits.

Aboveground resource‐acquisition traits showed different responses to competition treatments and soil moisture levels (Table 1; Figure 2). Under low soil moisture, plants under intraspecific competition showed higher SSL than plants under no‐competition and interspecific competition treatments (Figure 2a). ALM was higher under interspecific competition than under intraspecific and no‐competition treatments, whereas soil moisture had limited effects (Figure 2b). LA decreased under low soil moisture, while plants under interspecific competition showed greater LA than those under no‐competition treatments (Figure 2c). SLA generally increased with increasing soil moisture across competition treatments (Figure 2d).

**FIGURE 2:**
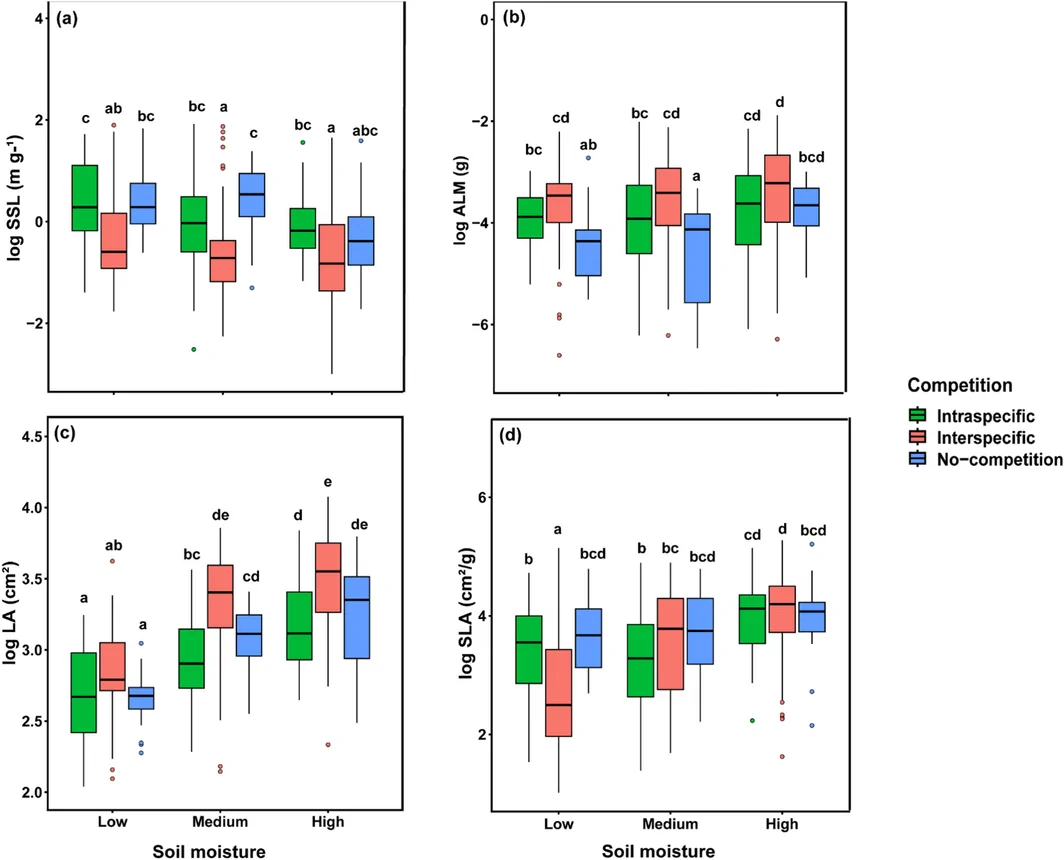
Effects of interspecific, intraspecific, and no‐competition treatments on aboveground functional traits related to resource acquisition across low, medium, and high soil moisture levels. Measured traits include (a) specific stem length (SSL, m g^−1^), (b) average leaf mass (ALM, g), (c) leaf area (LA, cm^2^), and (d) specific leaf area (SLA, cm^2^/g).

### 3.2 Response of Biomass Accumulation to Competition and Soil Moisture

Plant biomass showed different responses to competition treatments and soil moisture levels (Table 2; Figure 3). BGB was mainly affected by competition and soil moisture interactions. Under high and low soil moisture, plants with intraspecific competition showed higher BGB than plants with interspecific and no competition, whereas differences among competition treatments were limited under medium moisture. AGB increased under high soil moisture and was higher in plants with interspecific competition than in plants with intraspecific and no‐competition under soil moisture levels. TB showed a similar pattern, with greater biomass accumulation under high soil moisture and higher values in plants with interspecific competition compared with plants with intraspecific and no‐competition treatments.

**TABLE 2:** Summarizes the mixed‐effects model results for biomass accumulation and antioxidant/enzyme traits.

	BGB (log)	AGB (log)	TB (log)	CAT	MDA (log)	POD	SOD (log)
Random effects							
PotID	0.26	0.41	0.36	11.27	0.14	175.34	0.16
Species	0.16	0.33	0.25	3.20	0.00	220.99	0.04
Residual	0.80	0.59	0.53	51.72	0.35	776.02	0.32
Fixed effects							
Intercept	**−2.555**	−0.322	−0.147	**192.90**	**3.235**	**2253.0**	**0.381**
Competition (C): Inter	0.058	**1.194**	**1.141**	15.06	**0.202**	180.7	0.165
Competition (C): Intra	−0.306	**0.549**	**0.433**	−15.04	−0.051	−95.93	**0.324**
Soil moisture (SM): High	−0.438	**0.697**	**0.570**	−1.355	−0.023	−23.73	0.009
Soil moisture (SM): Low	0.105	−0.175	−0.087	16.012	**0.276**	**1003.4**	**0.602**
C: Inter × SM: High	0.210	**−0.532**	−0.457	−13.785	0.006	424.2	0.186
C: Intra × SM: High	**0.953**	−0.469	−0.009	25.193	**0.360**	−56.91	0.077
C: Inter × SM: Low	−0.033	0.005	−0.094	28.713	**−0.395**	487.02	−0.004
C: Intra × SM: low	**0.942**	−0.174	−0.013	**68.471**	**0.481**	210.63	**−0.480**
Explanatory power							
*R* ^2^m	0.112	0.254	0.273	0.226	0.243	0.383	0.284
*R* ^2^c	0.224	0.583	0.564	0.263	0.344	0.455	0.422

*Note:* Species and PotID were included as random effects, whereas competition treatments and soil moisture gradient were included as fixed effects. Competition was treated as a categorical factor with no competition as the reference level, and medium soil moisture was used as the reference category for the soil moisture gradient. All trait variables were transformed where necessary to meet model assumptions of normality and homoscedasticity. Model coefficients represent differences relative to these reference categories. Coefficients were evaluated using *t*‐tests with Satterthwaite‐adjusted degrees of freedom, and significant effects (*p* < 0.05) are shown in bold.

**FIGURE 3:**
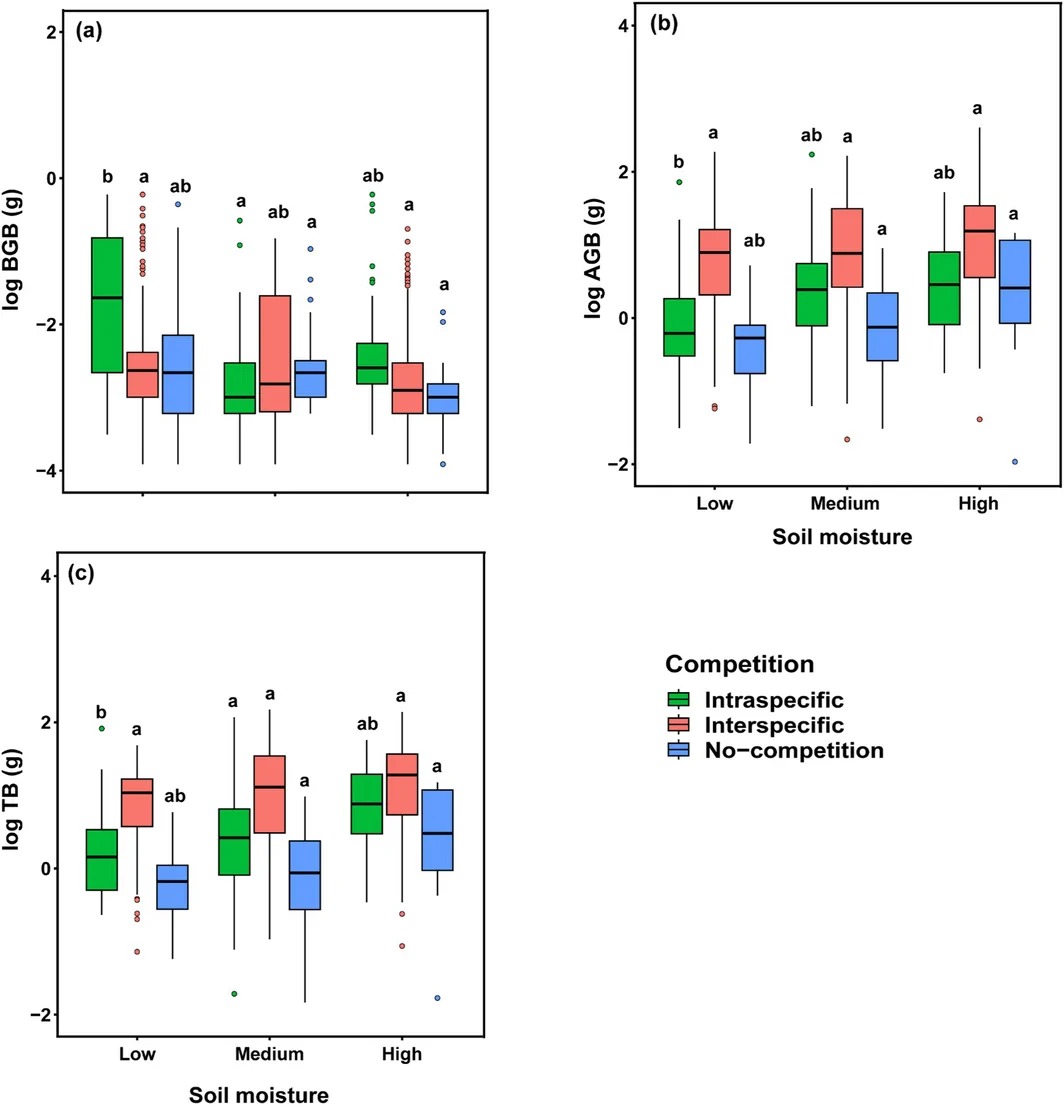
Effects of interspecific, intraspecific, and no‐competition treatments on biomass accumulation across low, medium, and high soil moisture levels. Biomass components include (a) belowground biomass (BGB, g), (b) aboveground biomass (AGB, g), and (c) total biomass (TB, g).

### 3.3 Response of Antioxidants Enzymes to Competition and Soil Moisture

Enzyme activities were significantly affected by competition treatments and soil moisture levels, with the strongest responses observed under low soil moisture compared with medium and high moisture levels (Table 2; Figure 4). Under low moisture conditions, CAT activity and MDA content were significantly higher under intraspecific competition than under interspecific and no‐competition treatments, whereas no significant differences among competition treatments were observed under medium and high moisture conditions. In contrast, POD and SOD activities were higher under interspecific competition than under intraspecific and no‐competition treatments, with the greatest differences observed under low soil moisture. Overall, enzyme activities showed different responses to competition treatments depending on soil moisture conditions.

**FIGURE 4:**
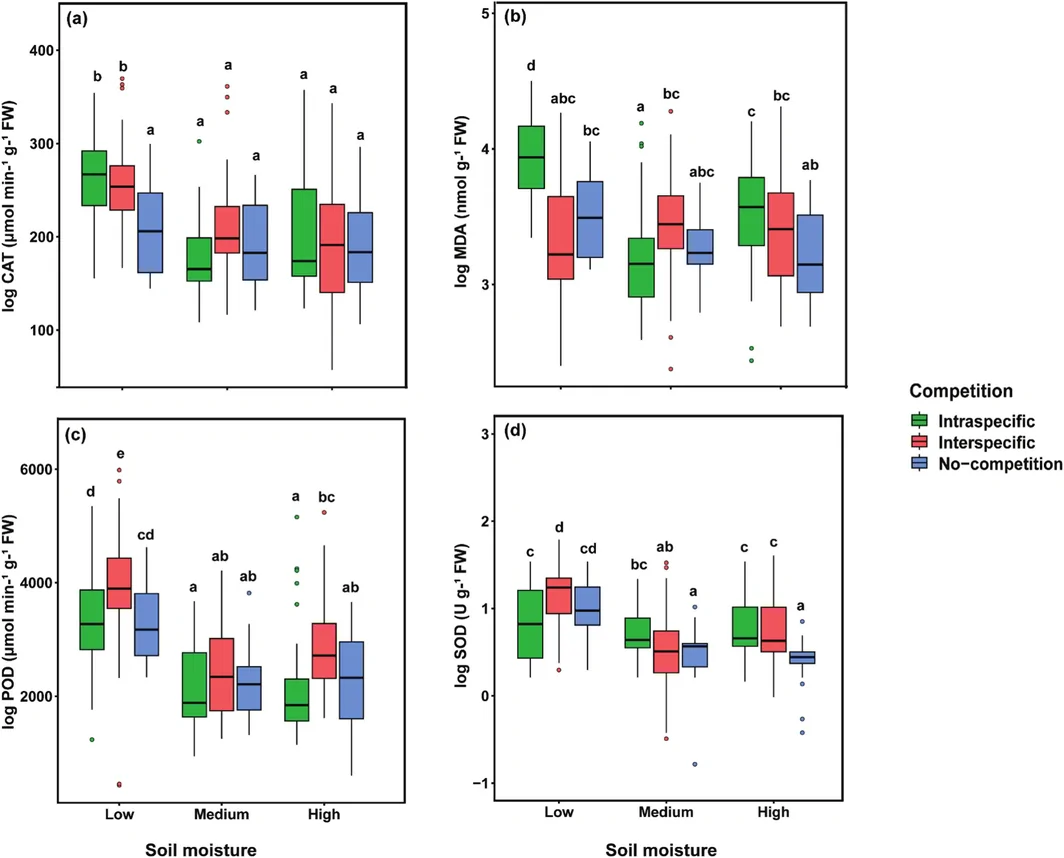
Effects of interspecific, intraspecific, and no‐competition treatments on antioxidant enzyme activities and oxidative stress indicators across low, medium, and high soil moisture levels. Measurements include (a) catalase activity (CAT, μmol min^−1^ g^−1^ FW), (b) malondialdehyde content (MDA, nmol g^−1^ FW), (c) peroxidase activity (POD, μmol min^−1^ g^−1^ FW), and (d) superoxide dismutase activity (SOD, U g^−1^ FW).

### 3.4 Species‐Specific Biomass Responses to Competition Treatments and Soil Moisture Gradients

Relative biomass change varied substantially among species, competition treatments, and soil moisture levels (Tables S2 and S3; Figure 5). Across soil moisture levels, interspecific competition generally resulted in greater biomass increases than intraspecific competition. Under low soil moisture, all species showed positive biomass changes under both competition treatments, although biomass increases were consistently greater under interspecific competition. *F. macrophylla* showed the largest biomass increases under both interspecific (282.8%) and intraspecific competition (116.3%), whereas *C. pallida* showed the weakest response, particularly under intraspecific competition (4.2%).

**FIGURE 5:**
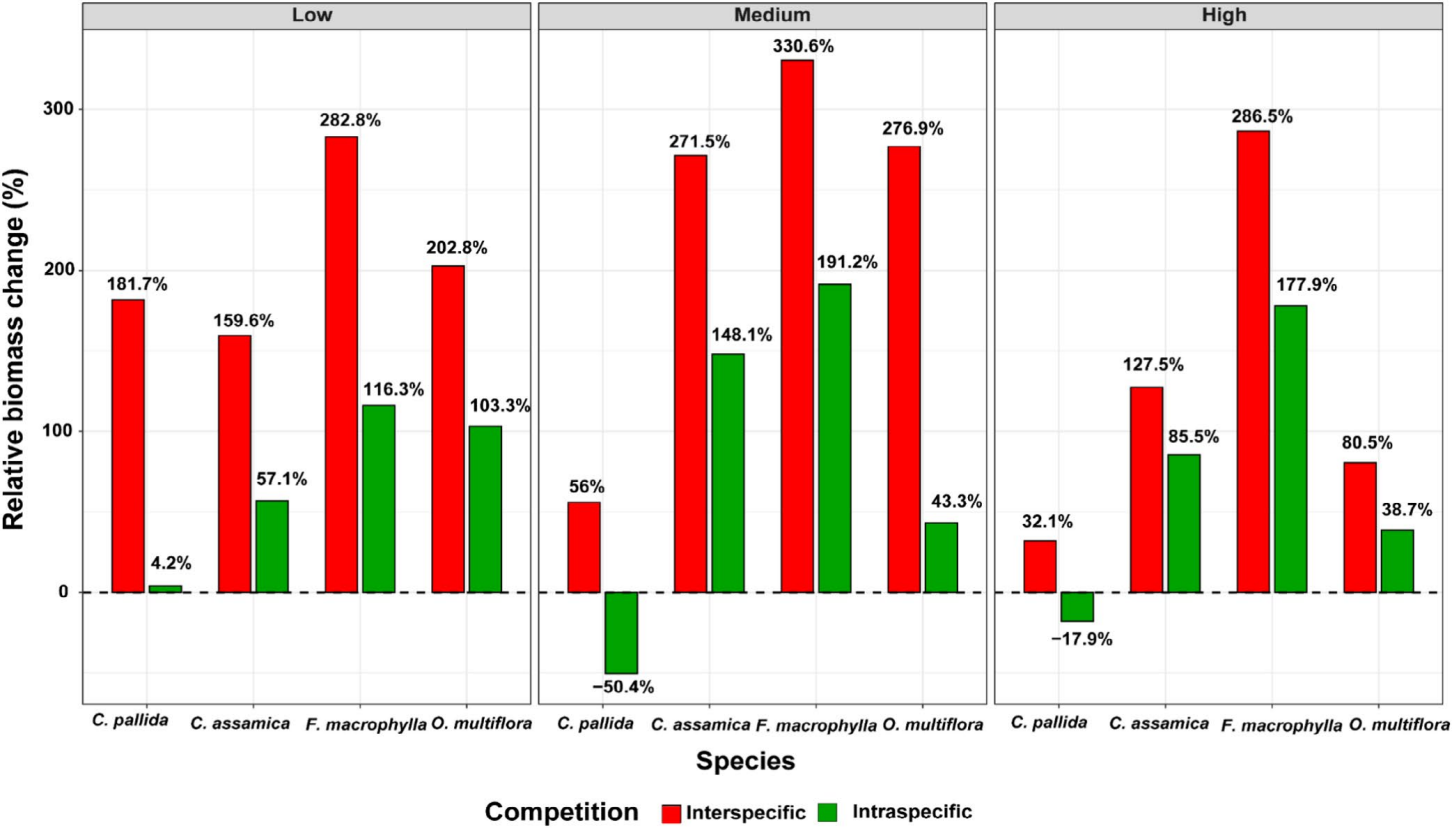
Relative biomass change (%) of four tropical legume species under intraspecific and interspecific competition across soil moisture gradients. Relative biomass change was calculated by comparing the total biomass (TB) of plants grown under intraspecific or interspecific competition with the mean total biomass of the same species grown without competition (TB_base), which served as the baseline. Positive values indicate higher biomass production relative to the no‐competition treatment, whereas negative values indicate reduced biomass compared with the no‐competition treatment.

Under medium soil moisture, interspecific competition resulted in positive biomass changes across all species, with the largest increase observed in *F. macrophylla* (330.6%), followed by *O. multiflora* (276.9%) and *C. assamica* (271.5%). In contrast, responses to intraspecific competition varied among species. *C. pallida* showed a negative biomass change (−50.4%), whereas *F. macrophylla* showed the largest increase (191.2%). Under high soil moisture, interspecific competition again resulted in positive biomass changes across all species, with the largest increase observed in *F. macrophylla* (286.5%). Intraspecific competition also resulted in positive biomass changes for most species, although *C. pallida* showed a negative response (−17.9%). Overall, *F. macrophylla* consistently showed the greatest biomass increases across competition treatments and soil moisture levels, whereas *C. pallida* showed the weakest biomass response, particularly under intraspecific competition (Table S3; Figure 5).

### 3.5 Relative Interaction Index (RII) Among Species Under Competition and Soil Moisture Gradients

RII values varied among species, competition treatments, and soil moisture levels (Table S1; Figure 6). BGB RII responses differed among species depending on competition treatments and soil moisture conditions. Under intraspecific competition, *F. macrophylla* showed negative BGB RII values under low and medium moisture conditions, whereas *C. assamica* and *O. multiflora* generally showed neutral or positive responses. Under interspecific competition, BGB RII values were generally higher than those under intraspecific competition, particularly in *C. assamica* and *F. macrophylla* . In contrast, *C. pallida* showed negative BGB RII values under low moisture under both competition treatments.

**FIGURE 6:**
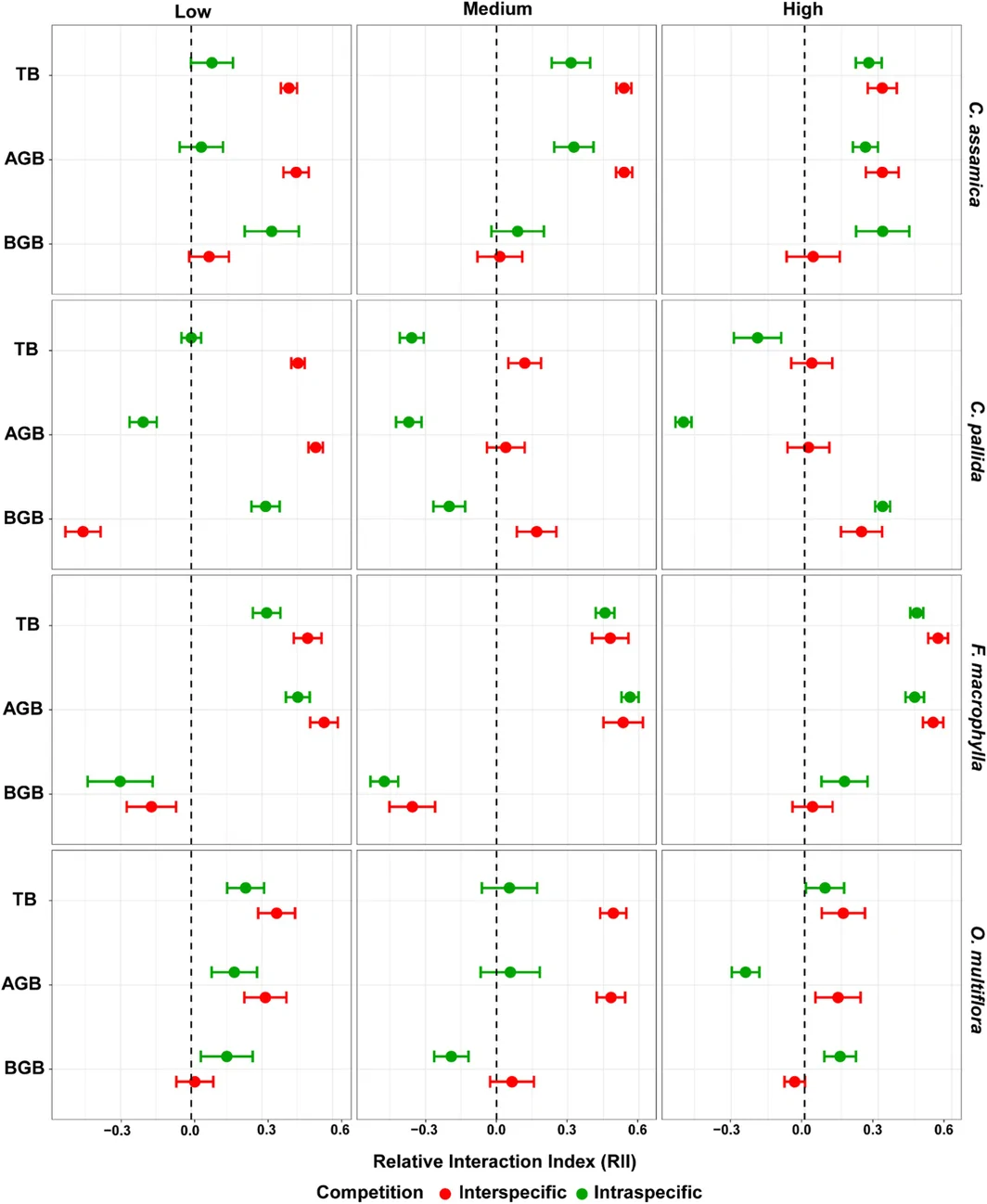
Relative interaction index (RII) values for belowground biomass (BGB), aboveground biomass (AGB), and total biomass (TB) of four tropical legume species under intraspecific and interspecific competition across soil moisture gradients. RII values range from −1 to +1, with negative values indicating reduced individual biomass relative to the no‐neighbor control (competitive effects), positive values indicating increased individual biomass relative to the no‐neighbor control (facilitative effects), and values close to zero indicating neutral interactions.

AGB showed mainly positive RII values under interspecific competition across species and soil moisture levels. The strongest positive responses were observed in *C. assamica* , *F. macrophylla* , and *O. multiflora* , whereas intraspecific competition resulted in weaker or near‐zero AGB RII values, particularly in *C. pallida* . Positive AGB RII values under interspecific competition were more pronounced under medium and high moisture conditions.

TB showed a similar pattern to AGB, with positive RII values under both interspecific and intraspecific competition across most species and soil moisture conditions. The largest positive TB responses were observed in *C. assamica* and *F. macrophylla* , particularly under high moisture conditions. In contrast, *C. pallida* showed negative TB RII values under intraspecific competition across soil moisture levels, whereas the other species generally maintained positive TB RII values under both competition treatments.

### 3.6 Effects of Competition and Soil Moisture on Relative Height Growth Rate

RHGR differed among species and showed variable responses to competition treatments and soil moisture levels (Figures S2 and S3). Across competition treatments, *C. assamica* maintained the highest RHGR under all neighbor treatments, with slightly higher values under intraspecific competition than under interspecific competition and no‐competition. In contrast, RHGR of *F. macrophylla* increased under intraspecific competition but was lower under no competition. The other two species showed relatively small differences among competition treatments. Across soil moisture levels, RHGR was generally higher under high moisture conditions, particularly for *C. assamica* and *F. macrophylla* , whereas RHGR was lower under low moisture. Overall, species differed in their growth responses to competition and soil moisture, indicating variation in their ability to maintain height growth under different environmental conditions.

### 3.7 Multivariate Trait Variation Under Competition and Soil Moisture Gradients

Principal component analysis (PCA) was conducted to evaluate overall variation in plant functional traits under different competition treatments and soil moisture levels. The first two principal components explained 39.0% of the total variation, with PC1 accounting for 21.5% and PC2 accounting for 17.5% (Tables S4–S6; Figure 7). The PCA ordination showed differences in trait distribution among soil moisture treatments, with plants under high moisture generally distributed toward the positive side of PC1, whereas plants under low moisture were mainly located toward the negative side of PC1 (Figure 7a). Trait contribution analysis showed that different functional traits contributed to the variation explained by PC1 and PC2 (Figure 7b). PC1 was mainly associated with biomass and growth‐related traits, with the highest contributions from RSR (17.29%), AGB (14.05%), LA (10.95%), NDM (9.77%), SSL (9.33%), and ALM (8.59%). In contrast, PC2 was mainly associated with leaf and physiological traits, with SLA (23.75%), AGB (16.64%), ALM (13.86%), SSL (8.06%), and POD (7.65%) showing the largest contributions. The loading patterns further showed the relationships between traits and the two principal components (Figure 7c). Growth‐related traits, including AGB, LA, NDM, NN, and ALM, showed positive loadings on PC1, whereas RSR and ARD showed negative loadings. For PC2, SLA showed the strongest positive loading, while antioxidant‐related traits, including SOD, POD, CAT, and MDA, showed negative loadings.

**FIGURE 7:**
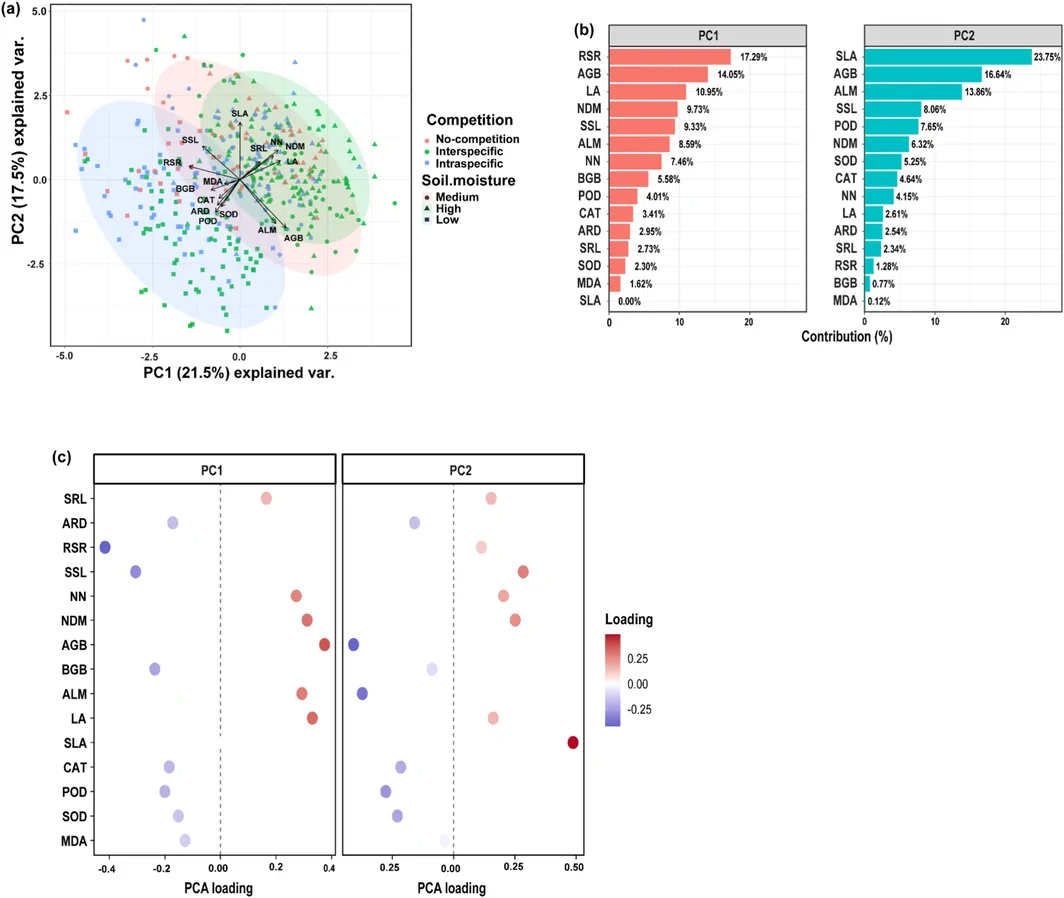
Multivariate variation in plant functional traits under different competition and soil moisture conditions. (a) PCA biplot showing the distribution of individual plants based on their functional trait profiles. Points represent individual plants and are colored according to competition treatments (no‐competition, interspecific competition, and intraspecific competition), while shaded ellipses represent different soil moisture levels (low, medium, and high). Trait vectors indicate the direction and strength of trait associations with PC1 and PC2, and axis labels show the percentage of total variation explained by each principal component. (b) Relative contribution of individual traits to PC1 and PC2. Bar lengths represent the percentage contribution of each trait to the variation explained by the corresponding principal component. (c) Loading coefficients of functional traits for PC1 and PC2. Points represent the loading values of each trait, with positive and negative values indicating the direction of association with the corresponding principal component. Trait variables showing strong skewness were log‐transformed before standardization and PCA, as described in the Methods. Traits are as follows: AGB, aboveground biomass; ALM, average leaf mass; ARD, average root diameter; BGB, belowground biomass; CAT, catalase activity; LA, leaf area; MDA, malondialdehyde content; NDM, nodule dry mass; NN, nodule number; POD, peroxidase activity; RSR, root‐to‐shoot ratio; SLA, specific leaf area; SOD, superoxide dismutase activity; SRL, specific root length; SSL, specific stem length.

## 4 Discussion

This study examined how intraspecific and interspecific interactions, together with soil moisture availability, influenced above‐ and belowground traits, physiological responses, and biomass accumulation in tropical legume species. We found that the effects of competition depended strongly on soil moisture, with intraspecific interactions promoting greater belowground biomass and resource‐acquisition traits, particularly under low soil moisture, whereas interspecific interactions enhanced aboveground biomass production. These contrasting responses were most pronounced across the moisture gradient, with interspecific interactions favoring aboveground growth under high moisture and intraspecific interactions strengthening belowground responses under water‐limited conditions. Overall, our findings demonstrate that competition type and soil moisture jointly shape species‐specific patterns of biomass accumulation and trait adjustment in tropical legume species.

### 4.1 Divergent Above‐ and Belowground Resource Acquisition Strategies Between Intraspecific and Interspecific Interactions

Our results demonstrate that tropical legume species adjusted their resource‐acquisition traits in response to soil moisture availability and competition treatment. Intraspecific interactions were associated with stronger belowground trait responses, whereas interspecific interactions promoted greater aboveground growth responses across moisture conditions. These contrasting patterns highlight the role of competition treatment in shaping plant allocation patterns under different moisture environments. SRL and ARD were generally higher under intraspecific competition, particularly under low soil moisture (Table 1; Figure 1), suggesting stronger root trait adjustments when plants interacted with conspecific individuals. Previous studies have shown that competition among individuals of the same species often involves greater overlap in resource requirements, which can increase belowground competition intensity (Robinson et al. 1999; Gersani et al. 2001; Montazeaud et al. 2025). Increased SRL and ARD under intraspecific competition may improve soil exploration and water acquisition per unit biomass, allowing plants to maintain resource uptake when water availability is limited (Novoplansky 2009; Farrior et al. 2013). Therefore, the stronger expression of root traits under intraspecific competition may reflect increased belowground resource acquisition associated with greater similarity in resource demand and niche overlap among conspecific individuals (Fruleux et al. 2016; Lorts and Lasky 2020; Minden and Olde Venterink 2019).

Under limited soil resources, theoretical models based on game theory predict excessive root proliferation as plants compete to preempt shared soil resources (Garrett 1968; Hardin 1968; Montazeaud et al. 2025). Such overinvestment may occur when the carbon costs of additional root growth exceed the benefits of resource acquisition, resulting in a belowground “tragedy of the commons” (TOC) (Anten and Vermeulen 2016; Gersani et al. 2001; Hardin 1968; Zhang et al. 1999). Empirical studies support this framework, showing that intense root competition can reduce aboveground performance, including yield losses associated with excessive topsoil root proliferation in wheat, soybean, and cereal systems (Gersani et al. 2001; Vain et al. 2023; Zhu et al. 2019). Consistent with these theoretical predictions, our results showed stronger belowground trait responses under intraspecific competition, particularly under low soil moisture. These findings suggest that conspecific competition under resource limitation may increase belowground allocation demands, with potential consequences for biomass production and plant performance in water‐limited environments (Montazeaud et al. 2025).

Variation in nodulation traits further supports the moisture‐dependent regulation of belowground acquisition responses (Table 1; Figure 1). Nodule number (NN) increased under high soil moisture, with plants under intraspecific competition showing greater nodule production than other competition treatments. In contrast, nodule dry mass (NDM) was mainly regulated by soil moisture, with greater nodule biomass under high moisture and reduced biomass under low moisture conditions. These changes in NN and NDM reflect plastic adjustments in legume–rhizobia symbiosis under changing environmental conditions (Marino et al. 2007; Zhang et al. 2020; Iqbal et al. 2022; Sher et al. 2026). Together, these patterns highlight the role of soil moisture and competition treatment in regulating belowground symbiotic nitrogen acquisition.

Aboveground traits showed contrasting responses to competition treatment and soil moisture availability. Interspecific competition increased LA and ALM, particularly under favorable moisture conditions (Table 1; Figure 2). This pattern suggests greater canopy development when plants interact with heterospecific individuals. Differences in resource demand between species may reduce competition intensity and allow greater allocation toward aboveground growth and light capture. SLA also increased with increasing soil moisture across competition treatments, reflecting greater leaf expansion under higher water availability (Bennett et al. 2016; Reich 2014; Wright et al. 2004; Zhu et al. 2015). Overall, these results demonstrate coordinated adjustments between above‐ and belowground resource‐acquisition traits. Plants did not show a uniform increase across all acquisition traits; instead, trait responses varied according to competition treatment and soil moisture availability (Rehling et al. 2021; Umaña et al. 2021; Zhang et al. 2021). Intraspecific interactions were mainly associated with stronger belowground trait responses under low soil moisture, whereas interspecific interactions promoted greater aboveground biomass and leaf development under favorable moisture conditions. Such context‐dependent trait adjustments may contribute to differences in species interactions and coexistence across heterogeneous environments (Novoplansky 2009; Lorts and Lasky 2020; Woo et al. 2024).

These patterns were further supported by PCA and correlation analyses, which revealed strong relationships among belowground traits, nodulation characteristics, and biomass‐related variables, while aboveground traits were more closely associated with overall plant growth responses (Figure 7; Table S5). These findings are consistent with the functional equilibrium hypothesis, which predicts that plants allocate resources toward organs responsible for acquiring the most limiting resources (Bloom et al. 1985). Collectively, our results support hypothesis (H1) by demonstrating that above‐ and belowground resource‐acquisition traits responded differently to soil moisture availability and competition treatments. Under low soil moisture and intraspecific competition, plants increased belowground acquisitive traits, including ARD and SRL, whereas interspecific interactions and higher soil moisture conditions were associated with greater expression of aboveground traits, including LA and SLA. Overall, tropical legumes showed strong trait plasticity, adjusting root and shoot characteristics in response to changing moisture availability and competitive environments.

### 4.2 Greater Aboveground Biomass Accumulation Under Interspecific Interactions Than Intraspecific Interactions Across Soil Moisture Gradients

Biomass responses across competition treatments and soil moisture gradients indicate that water availability was the primary environmental factor regulating plant growth, whereas neighbor identity influenced biomass allocation patterns. Across moisture conditions, intraspecific interactions consistently resulted in greater BGB than interspecific and no‐competition treatments (Table 2; Figure 3), suggesting stronger belowground competition among conspecific individuals due to greater similarity in resource requirements (Casper and Jackson 1997; Schenk 2006; Sher et al. 2024). However, greater root investment under intraspecific interactions was not accompanied by higher AGB or TB, indicating a trade‐off between belowground resource acquisition and aboveground growth. Thus, conspecific interactions may have promoted greater allocation toward root development as a response to stronger competitive pressure, enabling plants to maintain access to soil resources but limiting carbon allocation toward shoot production (File et al. 2012; Sher, Zheng, et al. 2025). In contrast, interspecific interactions resulted in greater AGB and TB across soil moisture conditions, suggesting that heterospecific neighbors reduced competitive constraints and promoted more efficient biomass production. Differences in functional traits and resource‐use patterns among species may reduce competition for shared resources and allow greater allocation toward aboveground growth (Kong et al. 2018; Rog et al. 2021; Beccari and Carmona 2024). Therefore, neighbor identity regulated the balance between competitive responses and biomass production, with conspecific interactions promoting greater belowground investment, whereas interspecific interactions favored greater whole‐plant productivity through more balanced resource allocation.

Relative biomass responses further demonstrated that the effects of neighbor identity varied among species, reflecting differences in their ability to respond to heterospecific and conspecific interactions under different moisture conditions (Figure 5; Tables S2 and S3). Across species, interspecific interactions generally resulted in greater biomass gains than intraspecific interactions, indicating that plants performed better when growing with heterospecific neighbors than with conspecific neighbors. However, the magnitude of these responses differed among species, with *F. macrophylla* , *O. multiflora* , and *C. assamica* showing stronger positive responses, whereas *C. pallida* were more sensitive to conspecific interactions, particularly under medium and high moisture conditions. This pattern suggests that the effects of neighbor identity become more pronounced when environmental stress is reduced and plants are able to express competitive differences under greater resource availability (Yamawo and Mukai 2020; Kopp et al. 2025). Species‐specific responses may further reflect differences in growth strategies. *C. pallida* and *C. assamica* showed characteristics of relatively acquisitive strategies, with rapid biomass accumulation under favorable conditions, whereas *F. macrophylla* and *O. multiflora* exhibited more stable growth responses that may reflect greater tolerance to environmental conditions (Ouédraogo et al. 2013; Chaturvedi et al. 2021; Yan et al. 2025). These findings indicate that the effects of neighbor identity are not consistent across species but depend on the interaction between species traits and environmental conditions.

Multivariate analysis further supported the importance of biomass production as a key component of plant responses to changing neighbor identity and soil moisture conditions. AGB contributed substantially to the variation explained by both PC1 and PC2, indicating that aboveground growth was closely associated with differences in plant performance across competitive and environmental conditions (Table S5; Figure 7b). Correlation analyses further revealed that plants modified biomass allocation strategies depending on interaction type. The positive relationship between plant height and BGB under intraspecific interactions (Figure S4) suggests that conspecific competition promoted greater root investment to support shoot development under stronger belowground competition. In contrast, the absence of this relationship under interspecific interactions indicates that plants could achieve greater aboveground growth without proportional increases in root biomass, reflecting more efficient biomass allocation. The negative relationship between SRL and BGB (Figure S5) further suggests a shift in root investment strategies under water limitation, where plants may favor thicker and more persistent roots rather than greater production of fine roots, improving resource retention under drought conditions (Hou et al. 2024). Similarly, relationships between shoot traits and biomass components further indicated that greater stem elongation was not necessarily associated with greater biomass accumulation, as SSL showed weak relationships with BGB but negative relationships with AGB and TB (Figure S6). In contrast, the strong positive association between AGB and TB highlights the dominant contribution of shoot production to overall plant productivity. These patterns are consistent with the functional equilibrium hypothesis, which predicts that plants preferentially allocate biomass toward organs involved in acquiring the most limiting resources (Levang‐Brilz and Biondini 2003; Larson and Funk 2016). Overall, these findings support the second hypothesis (H2) by demonstrating that neighbor identity regulated the balance between competitive effects and biomass performance across soil moisture gradients. Conspecific interactions generated stronger competitive responses, primarily through increased belowground investment, whereas interspecific interactions enhanced biomass production through more efficient allocation patterns and reduced competition for shared resources. However, the magnitude of these effects varied among species and depended on soil moisture conditions, indicating that plant interaction outcomes are jointly determined by neighbor identity, environmental availability, and species‐specific growth strategies.

### 4.3 Different Competitive Strategies Above‐ and Belowground Under Intraspecific and Interspecific Interactions

Our RII analysis revealed contrasting interactions between belowground and aboveground responses, demonstrating that the strength and direction of plant interactions were strongly influenced by neighbor identity and soil moisture conditions (Table S1; Figure 6). Belowground RII responses were more negative under intraspecific interactions, particularly for *F. macrophylla* under low soil moisture, indicating stronger competitive effects among conspecific neighbors. These negative responses likely result from greater overlap in root distribution and resource requirements, increasing competition for limited belowground resources (Casper and Jackson 1997; Schenk 2006; Sher et al. 2024; Sher, Zhang, and Zhang 2025). However, variation among species suggests that the intensity of belowground competition depends on species‐specific abilities to tolerate or respond to conspecific interactions.

In contrast, aboveground RII responses were generally more positive under interspecific interactions, whereas intraspecific interactions resulted in weaker or negative effects, particularly in *C. pallida* . This indicates that heterospecific neighbors altered the outcome of aboveground interactions, potentially through differences in canopy structure, growth form, and light acquisition strategies that reduce competitive interference (Michalet et al. 2014). Similarly, positive total biomass RII under interspecific interactions, especially under high moisture conditions, suggests that favorable environmental conditions can strengthen positive interaction outcomes, whereas conspecific interactions maintain stronger competitive effects. These patterns support the view that environmental conditions regulate the balance between competition and positive interactions, with soil moisture influencing the strength of interaction effects, while neighbor identity determines their direction (Michalet et al. 2023).

Several ecological mechanisms may explain the contrasting above‐ and belowground interaction patterns observed in this study. First, competitive interactions are strongly dependent on plant organs and the resources they acquire. Roots directly compete for limiting soil resources, including water and nutrients, and such competition becomes stronger when neighboring plants have similar root traits and resource requirements (Casper and Jackson 1997; Schenk 2006; Craine and Dybzinski 2013). In contrast, aboveground interactions may become less competitive or even positive when heterospecific neighbors differ in canopy structure, growth form, and light acquisition strategies, allowing greater resource complementarity among species (Brooker et al. 2008; Penanhoat et al. 2024; Sher et al. 2026). Second, neighbor identity regulates interaction outcomes through differences in functional similarity and resource overlap. Conspecific neighbors generally exhibit greater niche overlap and therefore stronger competition, whereas heterospecific neighbors may reduce competitive intensity through partial resource complementarity (Chesson 2000; Ashton et al. 2010). Third, soil moisture modifies the relative importance of competition and positive interactions. Under water limitation, belowground competition becomes stronger because plants compete directly for scarce soil water, whereas increased moisture availability may relax belowground constraints and allow aboveground complementarity to contribute more strongly to plant performance (Michalet et al. 2014). Finally, species‐specific growth strategies further shape interaction outcomes. Species with more acquisitive growth strategies may respond rapidly when resources are available, whereas species with more conservative strategies may maintain stable performance under environmental stress (Yan et al. 2025), resulting in different RII responses across species and moisture conditions. Together, these findings demonstrate that the balance between competition and positive interactions in tropical legume communities is determined by the interaction between neighbor identity, organ‐specific resource competition, soil moisture availability, and species‐specific strategies. Our results are consistent with previous studies showing that belowground competition is often strongest among conspecific neighbors, whereas interspecific interactions can promote positive effects through greater resource complementarity (Michalet et al. 2014; Fichtner et al. 2017). By integrating these mechanisms, our study provides insight into how mixed‐species communities maintain productivity and stability under variable soil moisture conditions.

## 5 Conclusion

This study shows that plant growth and competition responses in tropical legumes are mainly controlled by soil moisture conditions and the identity of neighboring plants. Together, these factors influence how plants allocate resources between roots and shoots and determine their growth strategies. Belowground, plants experienced stronger competition when growing with intraspecific neighbors, especially under low soil moisture, which promoted greater investment in roots. In contrast, interspecific interactions generally reduced belowground competition and allowed plants to allocate more resources to aboveground growth, particularly when soil moisture was sufficient, resulting in greater total biomass production. Overall, our findings demonstrate that plant–plant interactions are not fixed as either competition or facilitation but change depending on environmental conditions and neighbor identity. The strength and direction of these interactions were closely linked to plant trait adjustments, with different species adopting different strategies to acquire resources and maintain growth under changing soil moisture conditions.

This flexibility in plant responses may contribute to species coexistence by allowing plants to adjust their growth strategies under different environmental conditions. However, our findings should be considered within the limitations of a controlled glasshouse experiment. The use of pots, young plants, simplified soil conditions, and a relatively short experimental period may limit direct application of these results to mature tropical plant communities. Future studies should evaluate these interaction patterns under field conditions, with more complex soil communities and longer observation periods, to determine whether these moisture‐dependent responses persist at larger ecological scales. Such research will improve our understanding of how tropical plant communities respond to increasing climate stress and may provide useful guidance for mixed‐species restoration and sustainable ecosystem management.

## Author Contributions

**Jan Sher:** conceptualization (lead), data curation (lead), formal analysis (lead), investigation (lead), methodology (lead), software (lead), writing – original draft (lead). **Yaoqi Li:** writing – review and editing (equal). **Yun‐Bing Zhang:** project administration (equal), writing – review and editing (equal). **Mengguang Han:** writing – review and editing (equal). **Ghulam Mujtaba Shah:** writing – review and editing (equal). **Jiao‐Lin Zhang:** funding acquisition (equal), project administration (lead), resources (supporting), supervision (lead), visualization (supporting), writing – review and editing (equal).

## Funding

This research was supported by the Postdoctoral Fellowship Program of the Xishuangbanna Tropical Botanical Garden (XTBG), Chinese Academy of Sciences (CAS). The study also received financial support from the Foreigner Expert Project China (Y20240048), and the Caiyun Postdoctoral Program during the postdoctoral period. Additional funding was provided by the National Natural Science Foundation of China (Grant 32301308 and 32171507), the Yunnan Fundamental Research Projects (202401AT070230), and 14th Five‐Year Plan of the Xishuangbanna Tropical Botanical Garden, Chinese Academy of Sciences (XTBG‐1450101).

## Conflicts of Interest

The authors declare no conflicts of interest.

## Supporting information


**Data S1:** Raw experimental data on plant traits, growth, and competition under contrasting soil moisture conditions, used in the statistical analyses reported in this study.

**Table S1:** Relative Interaction Index (RII) values for belowground biomass (RII_BGB), aboveground biomass (RII_AGB), and total biomass (RII_TB) of four tropical legume species under different soil moisture conditions and neighbor treatments. For each species and soil moisture level, the RII was calculated using the mean individual biomass of plants grown without neighbors as the control reference. Positive RII values indicate facilitative effects, whereas negative values indicate competitive effects.
**Table S2:** The table represents the mean total biomass (TB_mean), baseline total biomass (TB_base), and relative change (%) in total biomass of all species under competition treatments.
**Table S3:** The table represents the mean total biomass (TB_mean), baseline total biomass (TB_base), and relative change (%) in total biomass of all species under soil moisture gradient treatments.
**Table S4:** Eigenvalues, percentage of variance explained, and cumulative variance (%) for the first 15 principal components (PCs) derived from principal component analysis (PCA) of measured plant traits.
**Table S5:** Loadings of 15 plant functional traits on the first five principal components (PC1–PC5) derived from principal component analysis (PCA). The PCA was conducted using standardized trait values across four legume species grown under intraspecific, interspecific, and no‐competition treatments. Traits are grouped into belowground resource acquisition (SRL, ARD, RSR, NN, and NDM), aboveground resource acquisition (SSL, ALM, LA, and SLA), biomass accumulation (BGB and AGB), and physiological responses (CAT, MDA, POD, and SOD). Higher absolute loading values indicate stronger associations between traits and the corresponding principal components, reflecting traits that contribute more strongly to multivariate trait variation.
**Table S6:** Correlation coefficients among 15 plant functional traits and the first five principal components (PC1–PC5) derived from principal component analysis (PCA). Significant correlations are indicated by *p* < 0.05 (*), *p* < 0.01 (**), and *p* < 0.001 (***). The table summarizes the relationships among functional traits and multivariate trait variation across species, soil moisture gradients, and neighbor treatments. Traits include belowground acquisition traits (SRL, ARD, RSR, NN, and NDM), aboveground acquisition traits (SSL, ALM, LA, and SLA), biomass accumulation traits (BGB and AGB), and physiological traits related to antioxidant activity (CAT, MDA, POD, and SOD). Positive and negative correlation coefficients indicate the direction and strength of trait associations with each principal component, with larger absolute values representing traits contributing more strongly to the corresponding multivariate trait axis.
**Figure S1:** Daily volumetric water content (VWC) in pots under low, medium, and high soil moisture treatments over the 200‐day experimental period. VWC was measured three to four times per week, both before and after irrigation on 3–4 days each week, to monitor soil water status. Soil moisture was maintained at the target levels throughout the experiment. Colored lines represent continuous VWC trends for each treatment, with high, medium, and low soil moisture levels shown in green, blue, and orange, respectively, highlighting differences in soil water availability over time.
**Figure S2:** Relative height growth rate (RHGR) of four species under different competition treatments. RHGR was calculated from the logarithmic change in plant height between the initial and final measurements over the 7‐month experimental period and expressed as monthly growth rate.
**Figure S3:** Relative height growth rate (RHGR) of four species under different soil moisture gradient treatments. RHGR was calculated from the logarithmic change in plant height between the initial and final measurements over the 7‐month experimental period and expressed as monthly growth rate.
**Figure S4:** Correlation plots showing pairwise relationships among plant height and biomass components under different competition treatments. Panels represent correlations between (a) plant height and belowground biomass, (b) plant height and aboveground biomass, (c) plant height and total biomass, (d) belowground biomass and aboveground biomass, (e) belowground biomass and total biomass, and (f) aboveground biomass and total biomass. Each point represents an individual pot, with competition treatments indicated by color: interspecific competition (red), intraspecific competition (green), and no competition (blue). Pearson correlation coefficients (*r*) and associated *p*‐values are shown in each panel to indicate the strength and significance of the relationships. The fitted regression lines and confidence intervals illustrate the direction and variation of relationships among plant height and biomass components across competition treatments.
**Figure S5:** Correlation plots showing relationships between specific root length (SRL) and biomass accumulation under different competition treatments. Panels represent correlations between (a) SRL and belowground biomass, (b) SRL and aboveground biomass, and (c) SRL and total biomass. Each point represents an individual pot, with competition treatments indicated by color: interspecific competition (red), intraspecific competition (green), and no competition (blue). Pearson correlation coefficients (*r*) and associated *p*‐values are shown in each panel to indicate the strength and significance of the relationships. The fitted regression lines and confidence intervals illustrate the direction and variation of SRL–biomass relationships across competition treatments.
**Figure S6:** Correlation plots showing pairwise relationships between aboveground resource acquisition traits and biomass accumulation under different competition treatments. Panels represent correlations between (a) specific stem length (SSL) and belowground biomass, (b) specific stem length (SSL) and aboveground biomass, (c) specific stem length (SSL) and total biomass, (d) specific leaf area (SLA) and belowground biomass, (e) specific leaf area (SLA) and aboveground biomass, and (f) specific leaf area (SLA) and total biomass. Each point represents an individual pot, with competition treatments indicated by color: interspecific competition (red), intraspecific competition (green), and no competition (blue). Pearson correlation coefficients (*r*) and associated *p*‐values are shown in each panel to indicate the strength and significance of the relationships. The fitted regression lines and confidence intervals illustrate the direction and variation of relationships between functional traits and biomass components across competition treatments.

## Data Availability

All datasets and statistical analysis results supporting the findings of this study are provided in the Supporting Information (Tables S1–S6; Figures S1–S6). The complete dataset used for statistical analyses is available as a CSV file and has been provided to the editors and reviewers during the peer‐review process. The dataset is publicly available in the Supporting Information provided with this article.

## References

[ece374586-bib-0001] Anderegg, L. D. L. , X. Loy , I. P. Markham , et al. 2021. “Aridity Drives Coordinated Trait Shifts but Not Decreased Trait Variance Across the Geographic Range of Eight Australian Trees.” New Phytologist 229: 1375–1387. 10.1111/nph.16795 32638379

[ece374586-bib-0002] Anten, N. P. R. , and P. J. Vermeulen . 2016. “Tragedies and Crops: Understanding Natural Selection to Improve Cropping Systems.” Trends in Ecology & Evolution 31: 429–439. 10.1016/j.tree.2016.02.010 27012675

[ece374586-bib-0003] Armas, C. , R. Ordiales , and F. I. Pugnaire . 2004. “Measuring Plant Interactions: A New Comparative Index.” Ecology 85: 2682–2686.

[ece374586-bib-0004] Armas, C. , S. Rodríguez‐Echeverría , and F. I. Pugnaire . 2011. “A Field Test of the Stress‐Gradient Hypothesis Along an Aridity Gradient.” Journal of Vegetation Science 22: 818–827.

[ece374586-bib-0005] Asefa, M. , S. J. Worthy , M. Cao , X. Song , Y. M. Lozano , and J. Yang . 2022. “Above‐ and Below‐Ground Plant Traits Are Not Consistent in Response to Drought and Competition Treatments.” Annals of Botany 130: 939–950. 10.1093/aob/mcac108 PMC9851322 36001733

[ece374586-bib-0006] Ashton, I. W. , A. E. Miller , W. D. Bowman , and K. N. Suding . 2010. “Niche Complementarity due to Plasticity in Resource Use: Plant Partitioning of Chemical N Forms.” Ecology 91: 3252–3260. 10.1890/09-1849.1 21141186

[ece374586-bib-0007] Bardgett, R. D. , L. Mommer , and F. T. De Vries . 2014. “Going Underground: Root Traits as Drivers of Ecosystem Processes.” Trends in Ecology & Evolution 29: 692–699. 10.1016/j.tree.2014.10.006 25459399

[ece374586-bib-0008] Bates, D. , M. Mächler , B. Bolker , and S. Walker . 2015. “Fitting Linear Mixed‐Effects Models Using lme4.” Journal of Statistical Software 67: 1–48.

[ece374586-bib-0009] Beccari, E. , and C. P. Carmona . 2024. “Aboveground and Belowground Sizes Are Aligned in the Unified Spectrum of Plant Form and Function.” Nature Communications 15: 9199. 10.1038/s41467-024-53180-x PMC11502772 39448582

[ece374586-bib-0010] Bennett, J. A. , K. Riibak , R. Tamme , R. J. Lewis , and M. Pärtel . 2016. “The Reciprocal Relationship Between Competition and Intraspecific Trait Variation.” Journal of Ecology 104: 1410–1420.

[ece374586-bib-0011] Berg, A. , and J. Sheffield . 2018. “Climate Change and Drought: The Soil Moisture Perspective.” Current Climate Change Reports 4: 180–191.

[ece374586-bib-0012] Bergmann, J. , A. Weigelt , F. van der Plas , et al. 2020. “The Fungal Collaboration Gradient Dominates the Root Economics Space in Plants.” Science Advances 6: eaba3756. 10.1126/sciadv.aba3756 PMC7458448 32937432

[ece374586-bib-0013] Bloom, A. J. , F. S. Chapin , and H. A. Mooney . 1985. “Resource Limitation in Plants—An Economic Analogy.” Annual Review of Ecology, Evolution, and Systematics 16: 363–392.

[ece374586-bib-0014] Brooker, R. W. , F. T. Maestre , R. M. Callaway , et al. 2008. “Facilitation in Plant Communities: The Past, the Present, and the Future.” Journal of Ecology 96: 18–34.

[ece374586-bib-0015] Brouwer, R. 1963. “Some Aspects of the Equilibrium Between Overground and Underground Plant Parts.” Jaarboek van het Instituut voor Biologisch en Scheikundig Onderzoek aan Landbouwgewassen 1963: 31–39.

[ece374586-bib-0016] Brunner, I. , C. Herzog , M. A. Dawes , M. Arend , and C. Sperisen . 2015. “How Tree Roots Respond to Drought.” Frontiers in Plant Science 6: 547. 10.3389/fpls.2015.00547 PMC4518277 26284083

[ece374586-bib-0017] Callaway, R. M. , and B. E. Mahall . 2007. “Family Roots.” Nature 448: 145–146. 10.1038/448145a 17625553

[ece374586-bib-0018] Cao, M. , X. Zou , M. Warren , and H. Zhu . 2006. “Tropical Forests of Xishuangbanna, China.” Biotropica 38: 306–309.

[ece374586-bib-0019] Carmona, C. P. , C. G. Bueno , A. Toussaint , et al. 2021. “Fine‐Root Traits in the Global Spectrum of Plant Form and Function.” Nature 597: 683–687. 10.1038/s41586-021-03871-y 34588667

[ece374586-bib-0020] Carmona, C. P. , F. De Bello , F. M. Azcárate , N. W. H. Mason , and B. Peco . 2019. “Trait Hierarchies and Intraspecific Variability Drive Competitive Interactions in Mediterranean Annual Plants.” Journal of Ecology 107: 2078–2089.

[ece374586-bib-0021] Casper, B. B. , and R. B. Jackson . 1997. “Plant Competition Underground.” Annual Review of Ecology, Evolution, and Systematics 28: 545–570.

[ece374586-bib-0022] Chaturvedi, R. K. , A. Tripathi , A. S. Raghubanshi , and J. S. Singh . 2021. “Functional Traits Indicate a Continuum of Tree Drought Strategies Across a Soil Water Availability Gradient in a Tropical Dry Forest.” Forest Ecology and Management 482: 118740.

[ece374586-bib-0023] Cheng, Y. , Y. Jiang , M. Li , et al. 2025. “Root Trait Responses of *Ligustrum lucidum* to Edaphic Stress Across Limestone and Sandy Shale Areas.” Plant and Soil 517: 635–653.

[ece374586-bib-0024] Chesson, P. 2000. “Mechanisms of Maintenance of Species Diversity.” Annual Review of Ecology, Evolution, and Systematics 31: 343–366.

[ece374586-bib-0025] Comas, L. H. , S. R. Becker , V. M. V. Cruz , P. F. Byrne , and D. A. Dierig . 2013. “Root Traits Contributing to Plant Productivity Under Drought.” Frontiers in Plant Science 4: 442. 10.3389/fpls.2013.00442 PMC3817922 24204374

[ece374586-bib-0026] Craine, J. M. , and R. Dybzinski . 2013. “Mechanisms of Plant Competition for Nutrients, Water and Light.” Functional Ecology 27: 833–840.

[ece374586-bib-0027] Deng, Y. , X. Jia , X. Sun , L. Liang , and J. Su . 2018. “Comparison of Jasmine Antioxidant System Responses to Different Degrees and Durations of Shade.” Acta Physiologiae Plantarum 40: 41.

[ece374586-bib-0028] Dixon, P. 2003. “VEGAN, a Package of R Functions for Community Ecology.” Journal of Vegetation Science 14: 927–930.

[ece374586-bib-0029] Donnelly, S. , M. Akin‐Fajiye , and L. H. Fraser . 2023. “Plant Provenance Can Influence the Impacts of Temperature and Moisture on Intraspecific Competition in *Pseudoroegneria spicata* .” Ecology and Evolution 13: e10603. 10.1002/ece3.10603 PMC10598250 37886429

[ece374586-bib-0030] Farrior, C. E. , D. Tilman , R. Dybzinski , P. B. Reich , S. A. Levin , and S. W. Pacala . 2013. “Resource Limitation in a Competitive Context Determines Complex Plant Responses to Experimental Resource Additions.” Ecology 94: 2505–2517. 10.1890/12-1548.1 24400502

[ece374586-bib-0031] Faucon, M.‐P. , D. Houben , and H. Lambers . 2017. “Plant Functional Traits: Soil and Ecosystem Services.” Trends in Plant Science 22: 385–394. 10.1016/j.tplants.2017.01.005 28209328

[ece374586-bib-0032] Fichtner, A. , W. Härdtle , Y. Li , H. Bruelheide , M. Kunz , and G. von Oheimb . 2017. “From Competition to Facilitation: How Tree Species Respond to Neighbourhood Diversity.” Ecology Letters 20: 892–900. 10.1111/ele.12786 28616871

[ece374586-bib-0033] File, A. L. , G. P. Murphy , and S. A. Dudley . 2012. “Fitness Consequences of Plants Growing With Siblings: Reconciling Kin Selection, Niche Partitioning and Competitive Ability.” Proceedings. Biological Sciences 279: 209–218. 10.1098/rspb.2011.1995 PMC3223689 22072602

[ece374586-bib-0034] Fitzpatrick, C. R. , Z. Mustafa , and J. Viliunas . 2019. “Soil Microbes Alter Plant Fitness Under Competition and Drought.” Journal of Evolutionary Biology 32: 438–450. 10.1111/jeb.13426 30739360

[ece374586-bib-0035] Foxx, A. J. , and F. Fort . 2019. “Root and Shoot Competition Lead to Contrasting Competitive Outcomes Under Water Stress: A Systematic Review and Meta‐Analysis.” PLoS One 14: e0220674. 10.1371/journal.pone.0220674 PMC6905553 31825953

[ece374586-bib-0036] Fruleux, A. , D. Bonal , and M.‐B. Bogeat‐Triboulot . 2016. “Interactive Effects of Competition and Water Availability on Above‐ and Below‐Ground Growth and Functional Traits of European Beech at Juvenile Level.” Forest Ecology and Management 382: 21–30.

[ece374586-bib-0037] Garrett, H. 1968. “The Tragedy of the Commons.” Science (80‐) 162: 1243–1248. 10.1126/science.162.3859.1243 17756331

[ece374586-bib-0038] Gersani, M. , J. S. Brown , E. E. O'Brien , G. M. Maina , and Z. Abramsky . 2001. “Tragedy of the Commons as a Result of Root Competition.” Journal of Ecology 89: 660–669.

[ece374586-bib-0039] Ghosh, U. , A. Chatterjee , and E. Bremer . 2018. “Determining the Moisture and Plant Effect on Nutrient Release, and Plant Nutrient Uptake Using Ion Exchange Resin Membrane.” Communications in Soil Science and Plant Analysis 49: 782–790.

[ece374586-bib-0040] Gratani, L. 2014. “Plant Phenotypic Plasticity in Response to Environmental Factors.” Advances in Botany 2014: 208747.

[ece374586-bib-0041] Hardin, G. 1968. “The Tragedy of the Commons: The Population Problem Has no Technical Solution; It Requires a Fundamental Extension in Morality.” Science (80‐) 162: 1243–1248. 5699198

[ece374586-bib-0042] Heilmeier, H. 2019. “Functional Traits Explaining Plant Responses to Past and Future Climate Changes.” Flora 254: 1–11.

[ece374586-bib-0043] Holmgren, M. , and M. Scheffer . 2010. “Strong Facilitation in Mild Environments: The Stress Gradient Hypothesis Revisited.” Journal of Ecology 98: 1269–1275.

[ece374586-bib-0044] Hou, J. , M. L. McCormack , P. B. Reich , et al. 2024. “Linking Fine Root Lifespan to Root Chemical and Morphological Traits—A Global Analysis.” Proceedings of the National Academy of Sciences of the United States of America 121: e2320623121. 10.1073/pnas.2320623121 PMC11032481 38607930

[ece374586-bib-0045] Iqbal, N. , V. O. Sadras , R. F. Denison , Y. Zhou , and M. D. Denton . 2022. “Clade‐Dependent Effects of Drought on Nitrogen Fixation and Its Components–Number, Size, and Activity of Nodules in Legumes.” Field Crops Research 284: 108586.

[ece374586-bib-0046] Jungklang, J. , K. Saengnil , and J. Uthaibutra . 2017. “Effects of Water‐Deficit Stress and Paclobutrazol on Growth, Relative Water Content, Electrolyte Leakage, Proline Content and Some Antioxidant Changes in *Curcuma alismatifolia* Gagnep. cv. Chiang Mai Pink.” Saudi Journal of Biological Sciences 24: 1505–1512. 10.1016/j.sjbs.2015.09.017 PMC6169545 30294219

[ece374586-bib-0047] Kebede, E. 2021. “Contribution, Utilization, and Improvement of Legumes‐Driven Biological Nitrogen Fixation in Agricultural Systems.” Frontiers in Sustainable Food Systems 5: 767998.

[ece374586-bib-0048] Kong, C.‐H. , S.‐Z. Zhang , Y.‐H. Li , et al. 2018. “Plant Neighbor Detection and Allelochemical Response Are Driven by Root‐Secreted Signaling Chemicals.” Nature Communications 9: 1–9. 10.1038/s41467-018-06429-1 PMC6155373 30250243

[ece374586-bib-0049] Kopp, E. B. , N. P. R. Anten , P. A. Niklaus , and S. E. Wuest . 2025. “Belowground Competition Increases Root Allocation in Agreement With Game‐Theoretical Predictions, but Only When Plants Simultaneously Compete Aboveground.” *bioRxiv*: 2001–2025. 10.1101/2025.01.29.635491.

[ece374586-bib-0050] Laliberté, E. 2017. “Below‐Ground Frontiers in Trait‐Based Plant Ecology.” New Phytologist 213: 1597–1603. 10.1111/nph.14247 27735077

[ece374586-bib-0051] Larson, J. E. , and J. L. Funk . 2016. “Seedling Root Responses to Soil Moisture and the Identification of a Belowground Trait Spectrum Across Three Growth Forms.” New Phytologist 210: 827–838. 10.1111/nph.13829 26765506

[ece374586-bib-0052] Levang‐Brilz, N. , and M. E. Biondini . 2003. “Growth Rate, Root Development and Nutrient Uptake of 55 Plant Species From the Great Plains Grasslands, USA.” Plant Ecology 165: 117–144.

[ece374586-bib-0053] Li, H. , Y. Ma , W. Liu , and W. Liu . 2012. “Soil Changes Induced by Rubber and Tea Plantation Establishment: Comparison With Tropical Rain Forest Soil in Xishuangbanna, SW China.” Environmental Management 50: 837–848. 10.1007/s00267-012-9942-2 22986585

[ece374586-bib-0054] Liu, W. , P. Li , W. Duan , and W. Liu . 2014. “Dry‐Season Water Utilization by Trees Growing on Thin Karst Soils in a Seasonal Tropical Rainforest of Xishuangbanna, Southwest China.” Ecohydrology 7: 927–935.

[ece374586-bib-0055] Liu, Z. , M. Zhao , H. Zhang , T. Ren , C. Liu , and N. He . 2023. “Divergent Response and Adaptation of Specific Leaf Area to Environmental Change at Different Spatio‐Temporal Scales Jointly Improve Plant Survival.” Global Change Biology 29: 1144–1159. 10.1111/gcb.16518 36349544

[ece374586-bib-0056] Lorts, C. M. , and J. R. Lasky . 2020. “Competition× Drought Interactions Change Phenotypic Plasticity and the Direction of Selection on Arabidopsis Traits.” New Phytologist 227: 1060–1072. 10.1111/nph.16593 32267968

[ece374586-bib-0057] Maestre, F. T. , R. M. Callaway , F. Valladares , and C. J. Lortie . 2009. “Refining the Stress‐Gradient Hypothesis for Competition and Facilitation in Plant Communities.” Journal of Ecology 97: 199–205.

[ece374586-bib-0058] Májeková, M. , T. Hájek , Á. J. Albert , et al. 2021. “Weak Coordination Between Leaf Drought Tolerance and Proxy Traits in Herbaceous Plants.” Functional Ecology 35: 1299–1311.

[ece374586-bib-0059] Marino, D. , P. Frendo , R. Ladrera , et al. 2007. “Nitrogen Fixation Control Under Drought Stress. Localized or Generalised Response?” Plant Physiology 143: 1968–1974. 10.1104/pp.106.097139 PMC1851803 17416644

[ece374586-bib-0060] Michalet, R. , F. Delerue , and P. Liancourt . 2023. “Disentangling the Effects of Biomass and Productivity in Plant Competition.” Ecology 104: e3851. 10.1002/ecy.3851 36054759

[ece374586-bib-0061] Michalet, R. , C. Schöb , C. J. Lortie , R. W. Brooker , and R. M. Callaway . 2014. “Partitioning Net Interactions Among Plants Along Altitudinal Gradients to Study Community Responses to Climate Change.” Functional Ecology 28: 75–86.

[ece374586-bib-0062] Minden, V. , and H. Olde Venterink . 2019. “Plant Traits and Species Interactions Along Gradients of N, P and K Availabilities.” Functional Ecology 33: 1611–1626.

[ece374586-bib-0063] Montazeaud, G. , P. Roumet , M. Lamboeuf , et al. 2025. “Mixing Varieties Mitigates Early Root Competition in Wheat Under Water and Nutrient Limitation.” Journal of Experimental Botany 76: 4171–4184. 10.1093/jxb/eraf163 PMC12448814 40292898

[ece374586-bib-0064] Nakagawa, S. , and H. Schielzeth . 2013. “A General and Simple Method for Obtaining R2 From Generalized Linear Mixed‐Effects Models.” Methods in Ecology and Evolution 4: 133–142.

[ece374586-bib-0065] Novoplansky, A. 2009. “Picking Battles Wisely: Plant Behaviour Under Competition.” Plant, Cell & Environment 32: 726–741. 10.1111/j.1365-3040.2009.01979.x 19389051

[ece374586-bib-0067] Oksanen, J. 2013. “Vegan: Ecological Diversity.” R Project 368: 1–11.

[ece374586-bib-0068] Ouédraogo, D. , F. Mortier , S. Gourlet‐Fleury , V. Freycon , and N. Picard . 2013. “Slow‐Growing Species Cope Best With Drought: Evidence From Long‐Term Measurements in a Tropical Semi‐Deciduous Moist Forest of Central Africa.” Journal of Ecology 101: 1459–1470.

[ece374586-bib-0069] Penanhoat, A. , N. Guerrero Ramirez , M. Aubry‐Kientz , et al. 2024. “Effect of Competition Intensity and Neighbor Identity on Architectural Traits of *Fagus sylvatica* .” Trees 38: 1177–1187.

[ece374586-bib-0070] Perez‐Harguindeguy, N. , S. Diaz , E. Garnier , et al. 2016. “Corrigendum to: New Handbook for Standardised Measurement of Plant Functional Traits Worldwide.” Australian Journal of Botany 64: 715–716.

[ece374586-bib-0071] Poorter, H. , and O. Nagel . 2000. “The Role of Biomass Allocation in the Growth Response of Plants to Different Levels of Light, CO_2_, Nutrients and Water: A Quantitative Review.” Australian Journal of Plant Physiology 27: 595–607.

[ece374586-bib-0072] R Core Team . 2022. R: A Language and Environment for Statistical Computing. R Foundation for Statistical Computing in Press.

[ece374586-bib-0073] Rajput, A. , Q. A. Panhwar , and H. Babar . 2025. “Role of Leguminous Crops by Enhancing Soil Fertility and Plant.” In Legume Crops for Food Security – Cultivation and Benefits. IntechOpen. In press.

[ece374586-bib-0074] Rao, A. S. , R. Chhawri , and A. Chauhan . 2024. “Ecosystem Responses to Global Environmental Challenges.” In Plant Functional Traits for Improving Productivity, 287. Springer Nature Singapore.

[ece374586-bib-0075] Rehling, F. , T. M. Sandner , and D. Matthies . 2021. “Biomass Partitioning in Response to Intraspecific Competition Depends on Nutrients and Species Characteristics: A Study of 43 Plant Species.” Journal of Ecology 109: 2219–2233.

[ece374586-bib-0076] Reich, P. B. 2014. “The World‐Wide ‘Fast–Slow’ Plant Economics Spectrum: A Traits Manifesto.” Journal of Ecology 102: 275–301.

[ece374586-bib-0077] Robinson, D. , A. Hodge , B. S. Griffiths , and A. H. Fitter . 1999. “Plant Root Proliferation in Nitrogen–Rich Patches Confers Competitive Advantage.” Proceedings of the Royal Society of London. Series B: Biological Sciences 266: 431–435.

[ece374586-bib-0078] Rog, I. , C. Tague , G. Jakoby , et al. 2021. “Interspecific Soil Water Partitioning as a Driver of Increased Productivity in a Diverse Mixed Mediterranean Forest.” Journal of Geophysical Research. Biogeosciences 126: e2021JG006382.

[ece374586-bib-0079] Saikia, P. , A. Nag , S. Anurag , S. Chatterjee , and M. L. Khan . 2020. “Tropical Legumes: Status, Distribution, Biology and Importance.” In The Plant Family Fabaceae: Biology and Physiological Responses to Environmental Stresses, 27–41. Springer.

[ece374586-bib-0080] Schenk, H. J. 2006. “Root Competition: Beyond Resource Depletion.” Journal of Ecology 94: 725–739.

[ece374586-bib-0081] Sher, J. , F. Bibi , G. Jan , K. W. Tomlinson , A. Ayaz , and W. Zaman . 2023. “Kin and Non‐Kin Connected Plants Benefit More Than Disconnected Kin and Non‐Kin Plants Under Nutrient‐Competitive Environments.” Plants 12: 487. 10.3390/plants12030487 PMC9920217 36771572

[ece374586-bib-0082] Sher, J. , N. Khan , and K. W. Tomlinson . 2024. “Plant Growth of *Chenopodium quinoa* (Willd) is Better When Growing With Kin Than With Non‐Kin Regardless of Soil Nutrient Conditions.” Plant Ecology 225: 153–161.

[ece374586-bib-0083] Sher, J. , Y.‐B. Zhang , G. M. Shah , S. P. Bonser , and J.‐L. Zhang . 2026. “Tropical Legume Plants Benefit More When Grown With Interspecific Plants Than Intraspecific Under Soil Phosphorus‐Limited Conditions.” Plant and Soil 526: 1–24.

[ece374586-bib-0084] Sher, J. , Y.‐B. Zhang , and J.‐L. Zhang . 2025. “Effects of Soil Fertility and Toxicity on the Performance of *Chenopodium quinoa* (Willd) Plants in Kin and Non‐Kin Interactions.” Plant Signaling & Behavior 20: 2591495. 10.1080/15592324.2025.2591495 PMC12674263 41324359

[ece374586-bib-0085] Sher, J. , Y. Zheng , J. H. Burns , G. Jan , and J.‐L. Zhang . 2025. “Kin Recognition in Plants‐An Ecological Perspective: An Overview of Plant Kin Recognition Under Different Resources, Consequences and Future Challenges.” Journal of Plant Interactions 20: 2548579.

[ece374586-bib-0086] Thomas, R. J. 1992. “The Role of the Legume in the Nitrogen Cycle of Productive and Sustainable Pastures.” Grass and Forage Science 47: 133–142.

[ece374586-bib-0087] Tomlinson, K. W. , F. J. Sterck , E. R. M. Barbosa , S. de Bie , H. H. T. Prins , and F. van Langevelde . 2019. “Seedling Growth of Savanna Tree Species From Three Continents Under Grass Competition and Nutrient Limitation in a Greenhouse Experiment.” Journal of Ecology 107: 1051–1066.

[ece374586-bib-0088] Tomlinson, K. W. , F. J. Sterck , F. Bongers , et al. 2012. “Biomass Partitioning and Root Morphology of Savanna Trees Across a Water Gradient.” Journal of Ecology 100: 1113–1121.

[ece374586-bib-0089] Umaña, M. N. , M. Cao , L. Lin , N. G. Swenson , and C. Zhang . 2021. “Trade‐Offs in Above‐ and Below‐Ground Biomass Allocation Influencing Seedling Growth in a Tropical Forest.” Journal of Ecology 109: 1184–1193.

[ece374586-bib-0090] Vain, S. , I. Tamm , Ü. Tamm , M. Annusver , and K. Zobel . 2023. “Negative Relationship Between Topsoil Root Production and Grain Yield in Oat and Barley.” Agriculture, Ecosystems and Environment 349: 108467.

[ece374586-bib-0091] Van Dyke, M. N. , J. M. Levine , and N. J. B. Kraft . 2022. “Small Rainfall Changes Drive Substantial Changes in Plant Coexistence.” Nature 611: 507–511. 10.1038/s41586-022-05391-9 36323782

[ece374586-bib-0092] Wang, C. , B. Fu , L. Zhang , and Z. Xu . 2019. “Soil Moisture–Plant Interactions: An Ecohydrological Review.” Journal of Soils and Sediments 19: 1–9.

[ece374586-bib-0093] Wang, Z. , and Y. Wei . 2022. “Physiological and Transcriptomic Analysis of Antioxidant Mechanisms in Sweet Sorghum Seedling Leaves in Response to Single and Combined Drought and Salinity Stress.” Journal of Plant Interactions 17: 1006–1016.

[ece374586-bib-0094] Wasson, A. P. , R. A. Richards , R. Chatrath , et al. 2012. “Traits and Selection Strategies to Improve Root Systems and Water Uptake in Water‐Limited Wheat Crops.” Journal of Experimental Botany 63: 3485–3498. 10.1093/jxb/ers111 22553286

[ece374586-bib-0095] Weemstra, M. , L. Mommer , E. J. W. Visser , et al. 2016. “Towards a Multidimensional Root Trait Framework: A Tree Root Review.” New Phytologist 211: 1159–1169. 10.1111/nph.14003 27174359

[ece374586-bib-0096] Weigelt, A. , L. Mommer , K. Andraczek , et al. 2021. “An Integrated Framework of Plant Form and Function: The Belowground Perspective.” New Phytologist 232: 42–59. 10.1111/nph.17590 34197626

[ece374586-bib-0097] Woo, S. , T. Kim , Y. Kim , S. Jeong , and E. Kim . 2024. “Inter‐ and Intraspecific Competition in Invasive *Lactuca serriola* and Co‐Occurring Weedy Plant Species.” Ecology and Evolution 14: e70496. 10.1002/ece3.70496 PMC11522139 39478980

[ece374586-bib-0098] Wright, I. J. , P. B. Reich , M. Westoby , et al. 2004. “The Worldwide Leaf Economics Spectrum.” Nature 428: 821–827. 10.1038/nature02403 15103368

[ece374586-bib-0099] Yamawo, A. , and H. Mukai . 2020. “Outcome of Interspecific Competition Depends on Genotype of Conspecific Neighbours.” Oecologia 193: 415–423. 10.1007/s00442-020-04694-w 32577823

[ece374586-bib-0100] Yan, P. , N. He , M. Fernández‐Martínez , et al. 2025. “Plant Acquisitive Strategies Promote Resistance and Temporal Stability of Semiarid Grasslands.” Ecology Letters 28: e70110. 10.1111/ele.70110 PMC11967160 40178069

[ece374586-bib-0101] Yang, J. , G. Shen , X. Wang , and C. P. Carmona . 2025. “Competition‐Induced Trait Variability Obscures Trait–Growth Relationships of Tree Seedlings.” Ecology Letters 28: e70259. 10.1111/ele.70259 41206875

[ece374586-bib-0102] Younginger, B. S. , D. Sirová , M. B. Cruzan , and D. J. Ballhorn . 2017. “Is Biomass a Reliable Estimate of Plant Fitness?” Applications in Plant Sciences 5: 1600094. 10.3732/apps.1600094 PMC5315378 28224055

[ece374586-bib-0103] Yu, Q. , and Z. Rengel . 1999. “Drought and Salinity Differentially Influence Activities of Superoxide Dismutases in Narrow‐Leafed Lupins.” Plant Science 142: 1–11.

[ece374586-bib-0104] Zhang, D. , Y. Peng , F. Li , et al. 2021. “Above‐ and Below‐Ground Resource Acquisition Strategies Determine Plant Species Responses to Nitrogen Enrichment.” Annals of Botany 128: 31–44. 10.1093/aob/mcab032 PMC8318111 33630994

[ece374586-bib-0105] Zhang, D.‐Y. , G.‐J. Sun , and X.‐H. Jiang . 1999. “Donald's Ideotype and Growth Redundancy: A Game Theoretical Analysis.” Field Crops Research 61: 179–187.

[ece374586-bib-0106] Zhang, J. , and M. B. Kirkham . 1994. “Drought‐Stress‐Induced Changes in Activities of Superoxide Dismutase, Catalase, and Peroxidase in Wheat Species.” Plant & Cell Physiology 35: 785–791.

[ece374586-bib-0107] Zhang, J. , K. Yu , M. Reichstein , et al. 2026. “Trait Space and Compactness: A New Perspective on Explaining Variations in Primary Productivity.” Ecology 107: e70278. 10.1002/ecy.70278 41531259

[ece374586-bib-0108] Zhang, R. , and K. Tielbörger . 2019. “Facilitation From an Intraspecific Perspective–Stress Tolerance Determines Facilitative Effect and Response in Plants.” New Phytologist 221: 2203–2212. 10.1111/nph.15528 30298569

[ece374586-bib-0109] Zhang, X. , L. Wang , J. Li , R. T. Batstone , and M. E. Frederickson . 2020. “ *Medicago truncatula* Adjusts Root Proliferation, Nodule Formation, and Partner Choice in Response to Local N Heterogeneity.” Plant and Soil 450: 417–428.

[ece374586-bib-0110] Zhu, J. , W. van der Werf , N. P. R. Anten , J. Vos , and J. B. Evers . 2015. “The Contribution of Phenotypic Plasticity to Complementary Light Capture in Plant Mixtures.” New Phytologist 207: 1213–1222. 10.1111/nph.13416 25898768

[ece374586-bib-0111] Zhu, Y.‐H. , J. Weiner , and F.‐M. Li . 2019. “Root Proliferation in Response to Neighbouring Roots in Wheat ( *Triticum aestivum* ).” Basic and Applied Ecology 39: 10–14.

