# Supplementary material for: Divergent Root and Shoot Trait Strategies Shape Competition–Facilitation Shifts Between Conspecific and Heterospecific Neighbors Under Contrasting Soil Moisture Conditions
Source: Ecol Evol. 2026 Oct 23;16(10):e74586. doi: 10.1002/ece3.74586 (PMC13694201; doi:10.1002/ece3.74586)
Supplement: Supplementary file 1 [file ECE3-16-e74586-s001.docx]

**Divergent Root and Shoot Trait Strategies Shape Competition–Facilitation Shifts Between Conspecific and Heterospecific Neighbors Under Contrasting Soil Moisture Conditions**

Jan Sher^1,2^, Yaoqi Li^3^, Yun-Bing Zhang^1,2^, Mengguang Han^4^, Ghulam Mujtaba Shah^5^, Jiao-Lin Zhang^1,2*^

^1^Yunnan Key Laboratory of Forest Ecosystem Stability and Global Change, Xishuangbanna Tropical Botanical Garden, Chinese Academy of Sciences, Mengla, Yunnan, 666303, China

^2^Laboratory of Tropical Forest Ecology, Xishuangbanna Tropical Botanical Garden, Chinese Academy of Sciences, Mengla, Yunnan, 666303, China

^3^Department of Health and Environmental Sciences, School of Science, Xi'an Jiaotong-Liverpool University, Suzhou 215124, China

^4^Ministry of Education Key Laboratory of Sustainable Forest Ecosystem Management, Northeast Asia Biodiversity Research Center, and School of Ecology, Northeast Forestry University, Harbin, China

^5^Department of Botany, Hazara University Mansehra, Dhodial, Pakistan

Professor. Jiao-Lin Zhang (corresponding author)
Telephone: 00866918715462

Fax: 00866918715070

E-mail: (zjl@xtbg.org.cn)

**Table S1:** Relative Interaction Index (RII) values for belowground biomass (RII_BGB), aboveground biomass (RII_AGB), and total biomass (RII_TB) of four tropical legume species under different soil moisture conditions and neighbor treatments. For each species and soil moisture level, the RII was calculated using the mean individual biomass of plants grown without neighbors as the control reference. Positive RII values indicate facilitative effects, whereas negative values indicate competitive effects.

| **Species** | **Soil moisture** | **Competition** | **RII_BGB** | **RII_AGB** | **RII_TB** |
| --- | --- | --- | --- | --- | --- |
| *C. pallida* | Low | Interspecific | 0.019 | 0.323 | 0.309 |
| *C. pallida* | Low | Intraspecific | 0.712 | -0.427 | -0.16 |
| *C. pallida* | Medium | Interspecific | 0.168 | 0.038 | 0.118 |
| *C. pallida* | Medium | Intraspecific | -0.199 | -0.370 | -0.358 |
| *C. pallida* | High | Interspecific | -0.024 | 0.326 | 0.327 |
| *C. pallida* | High | Intraspecific | 0.027 | -0.175 | 0.121 |
| *C. assamica* | Low | Interspecific | -0.011 | 0.427 | 0.392 |
| *C. assamica* | Low | Intraspecific | -0.266 | 0.021 | 0.063 |
| *C. assamica* | Medium | Interspecific | 0.0134 | 0.537 | 0.536 |
| *C. assamica* | Medium | Interspecific | 0.088 | 0.325 | 0.313 |
| *C. assamica* | High | Interspecific | 0.0135 | 0.505 | 0.499 |
| *C. assamica* | High | Intraspecific | 0.297 | 0.458 | 0.459 |
| *F. macrophylla* | Low | Interspecific | -0.032 | 0.468 | 0.472 |
| *F. macrophylla* | Low | Intraspecific | -0.177 | 0.336 | 0.294 |
| *F. macrophylla* | Medium | Interspecific | -0.355 | 0.533 | 0.479 |
| *F. macrophylla* | Medium | Intraspecific | -0.473 | 0.562 | 0.457 |
| *F. macrophylla* | High | Interspecific | -0.404 | 0.664 | 0.608 |
| *F. macrophylla* | High | Intraspecific | -0.295 | 0.608 | 0.532 |
| *O. multiflora* | Low | Interspecific | 0.014 | 0.159 | 0.219 |
| *O. multiflora* | Low | Intraspecific | -0.150 | 0.010 | 0.069 |
| *O. multiflora* | Medium | Interspecific | 0.064 | 0.482 | 0.491 |
| *O. multiflora* | Medium | Intraspecific | -0.190 | 0.057 | 0.054 |
| *O. multiflora* | High | Interspecific | -0.088 | 0.373 | 0.382 |
| *O. multiflora* | High | Intraspecific | 0.098 | 0.042 | 0.334 |

**Table S2:** The table represents the mean total biomass (TB_mean), baseline total biomass (TB_base), and relative change (%) in total biomass of all species under competition treatments.

| Species | Competition | TB_mean | TB_base | Relative change% |
| --- | --- | --- | --- | --- |
| *C. pallida* | Interspecific | 2.935 | 1.759 | 66.83 |
| *C. pallida* | Intraspecific | 1.365 | 1.759 | -22.41 |
| *C. assamica* | Interspecific | 4.375 | 1.582 | 176.59 |
| *C. assamica* | Intraspecific | 3.093 | 1.582 | 95.56 |
| *F. macrophylla* | Interspecific | 2.771 | 0.694 | 299.32 |
| *F. macrophylla* | Intraspecific | 1.830 | 0.694 | 163.74 |
| *O. multiflora* | Interspecific | 2.809 | 1.072 | 161.99 |
| *O. multiflora* | Intraspecific | 1.641 | 1.072 | 53.10 |

**Table S3:** The table represents the mean total biomass (TB_mean), baseline total biomass (TB_base), and relative change (%) in total biomass of all species under soil moisture gradient treatments.

| Species | Soil moisture | TB_mean | TB_base | Relative change% |
| --- | --- | --- | --- | --- |
| *C. pallida* | High | 3.165 | 1.572 | 101.30 |
| *C. pallida* | Low | 1.948 | 1.572 | 23.89 |
| *C. assamica* | High | 4.178 | 3.879 | 7.69 |
| *C. assamica* | Low | 2.553 | 3.879 | -34.19 |
| *F. macrophylla* | High | 2.478 | 2.208 | 12.26 |
| *F. macrophylla* | Low | 1.768 | 2.208 | -19.88 |
| *O. multiflora* | High | 2.531 | 2.392 | 5.81 |
| *O. multiflora* | Low | 1.558 | 2.392 | -34.87 |

**Table S4:** Eigenvalues, percentage of variance explained, and cumulative variance (%) for the first 15 principal components (PCs) derived from principal component analysis (PCA) of measured plant traits.

| PC Number | Eigenvalue | Variance explained (%) | Cumulative variance (%) |
| --- | --- | --- | --- |
| PC1 | 3.23 | 21.548 | 21.548 |
| PC2 | 2.621 | 17.478 | 39.027 |
| PC3 | 2.122 | 14.146 | 53.173 |
| PC4 | 1.071 | 7.140 | 60.313 |
| PC5 | 1.014 | 6.760 | 67.074 |
| PC6 | 0.865 | 5.771 | 72.845 |
| PC7 | 0.783 | 5.221 | 78.067 |
| PC8 | 0.706 | 4.709 | 82.776 |
| PC9 | 0.630 | 4.201 | 86.978 |
| PC10 | 0.600 | 4.003 | 90.981 |
| PC11 | 0.538 | 3.592 | 94.574 |
| PC12 | 0.377 | 2.519 | 97.093 |
| PC13 | 0.279 | 1.863 | 98.957 |
| PC14 | 0.119 | 0.799 | 99.607 |
| PC15 | 0.036 | 0.242 | 100 |

**Table S5:** Loadings of 15 plant functional traits on the first five principal components (PC1–PC5) derived from principal component analysis (PCA). The PCA was conducted using standardized trait values across four legume species grown under intraspecific, interspecific, and no-competition treatments. Traits are grouped into belowground resource acquisition (SRL, ARD, RSR, NN, and NDM), aboveground resource acquisition (SSL, ALM, LA, and SLA), biomass accumulation (BGB and AGB), and physiological responses (CAT, MDA, POD, and SOD). Higher absolute loading values indicate stronger associations between traits and the corresponding principal components, reflecting traits that contribute more strongly to multivariate trait variation.

| **Trait** | **Group** | **PC1** | **PC2** | **PC3** | **PC4** | **PC5** |
| --- | --- | --- | --- | --- | --- | --- |
|  |  | 21.5 | 17.5 | 10 | 8.8 | 6.6 |
| SRL | Belowground Resource Acquisition trait | 0.165 | 0.153 | -0.575 | -0.188 | 0.052 |
| ARD | Belowground Resource Acquisition trait | -0.171 | -0.159 | -0.118 | -0.277 | -0.584 |
| RSR | Root allocation trait | -0.415 | 0.113 | 0.385 | 0.095 | -0.051 |
| NN | Symbiotic nitrogen acquisition trait | 0.273 | 0.203 | 0.220 | -0.279 | 0.049 |
| NDM | Symbiotic nitrogen acquisition trait | 0.311 | 0.251 | 0.143 | -0.014 | -0.034 |
| SSL | Aboveground resource-acquisition trait | -0.305 | 0.283 | -0.187 | -0.085 | -0.056 |
| ALM | Biomass allocation traits | 0.293 | -0.372 | 0.107 | -0.142 | -0.077 |
| LA | Aboveground resource-acquisition trait | 0.330 | 0.161 | 0.267 | -0.057 | 0.188 |
| SLA | Aboveground resource-acquisition trait | 0.001 | 0.487 | 0.146 | -0.109 | 0.257 |
| BGB | Biomass Accumulation | -0.236 | -0.087 | 0.492 | 0.054 | -0.125 |
| AGB | Biomass Accumulation | 0.374 | -0.407 | 0.161 | 0.013 | -0.015 |
| CAT | Antioxidant defense and oxidative stress indicator | -0.184 | -0.215 | -0.008 | -0.391 | 0.163 |
| MDA | Antioxidant defense and oxidative stress indicator | -0.127 | -0.035 | 0.1393 | -0.768 | 0.124 |
| POD | Antioxidant defense and oxidative stress indicator | -0.200 | -0.276 | -0.070 | 0.099 | 0.421 |
| SOD | Antioxidant defense and oxidative stress indicator | -0.151 | -0.229 | -0.068 | 0.024 | 0.550 |

**Table S6:** Correlation coefficients among 15 plant functional traits and the first five principal components (PC1–PC5) derived from principal component analysis (PCA). Significant correlations are indicated by *p* < 0.05 (^*^), *p* < 0.01 (^**^) and *p < 0.001* (*^***^*). The table summarizes the relationships among functional traits and multivariate trait variation across species, soil moisture gradients, and neighbor treatments. Traits include belowground acquisition traits (SRL, ARD, RSR, NN, and NDM), aboveground acquisition traits (SSL, ALM, LA, and SLA), biomass accumulation traits (BGB and AGB), and physiological traits related to antioxidant activity (CAT, MDA, POD, and SOD). Positive and negative correlation coefficients indicate the direction and strength of trait associations with each principal component, with larger absolute values representing traits contributing more strongly to the corresponding multivariate trait axis.

| **Trait** | **PC1** | **PC2** | **PC3** | **PC4** | **PC5** |
| --- | --- | --- | --- | --- | --- |
| SRL | 0.297^***^ | 0.248^***^ | 0.547*** | -0.195*** | 0.052 |
| ARD | -0.309*** | -0.258*** | -0.173*** | -0.288*** | -0.588*** |
| RSR | -0.747*** | 0.183*** | 0.562*** | 0.098* | -0.052 |
| NN | 0.491*** | 0.330*** | 0.322*** | -0.290*** | 0.049 |
| NDM | 0.561*** | 0.407*** | 0.210*** | -0.015 | -0.035 |
| SSL | -0.549*** | 0.460*** | -0.273*** | -0.088 | -0.057 |
| ALM | 0.527*** | -0.603*** | 0.156** | -0.148** | -0.078 |
| LA | 0.595*** | 0.262*** | 0.389*** | -0.059 | 0.190*** |
| SLA | 0.002 | 0.789*** | 0.213*** | -0.113* | 0.259*** |
| BGB | -0.425*** | -0.142** | 0.717*** | 0.057 | -0.126** |
| AGB | 0.674*** | -0.661*** | 0.235*** | 0.014 | -0.016 |
| CAT | 0.332*** | -0.349*** | -0.012 | -0.405*** | 0.164*** |
| MDA | -0.229*** | -0.057 | 0.203*** | -0.795*** | 0.125* |
| POD | -0.360*** | -0.448*** | -0.102* | 0.103* | 0.425*** |
| SOD | -0.273*** | -0.371*** | -0.100* | 0.025 | 0.554*** |

**Figure S1.** Daily volumetric water content (VWC) in pots under low, medium, and high soil moisture treatments over the 200-day experimental period. VWC was measured three to four times per week, both before and after irrigation on three to four days each week, to monitor soil water status. Soil moisture was maintained at the target levels throughout the experiment. Colored lines represent continuous VWC trends for each treatment, with high, medium, and low soil moisture levels shown in green, blue, and orange, respectively, highlighting differences in soil water availability over time.

**Figure S2.** Relative height growth rate (RHGR) of four species under different competition treatments. RHGR was calculated from the logarithmic change in plant height between the initial and final measurements over the seven-month experimental period and expressed as monthly growth rate.

**Figure S3.** Relative height growth rate (RHGR) of four species under different soil moisture gradient treatments. RHGR was calculated from the logarithmic change in plant height between the initial and final measurements over the seven-month experimental period and expressed as monthly growth rate.

**Figure S4.** Correlation plots showing pairwise relationships among plant height and biomass components under different competition treatments. Panels represent correlations between (a) plant height and below-ground biomass, (b) plant height and above-ground biomass, (c) plant height and total biomass, (d) below-ground biomass and above-ground biomass, (e) below-ground biomass and total biomass, and (f) above-ground biomass and total biomass. Each point represents an individual pot, with competition treatments indicated by color: interspecific competition (red), intraspecific competition (green), and no competition (blue). Pearson correlation coefficients (*r*) and associated *p*-values are shown in each panel to indicate the strength and significance of the relationships. The fitted regression lines and confidence intervals illustrate the direction and variation of relationships among plant height and biomass components across competition treatments.

**Figure S5.** Correlation plots showing relationships between specific root length (SRL) and biomass accumulation under different competition treatments. Panels represent correlations between (a) SRL and below-ground biomass, (b) SRL and above-ground biomass, and (c) SRL and total biomass. Each point represents an individual pot, with competition treatments indicated by color: interspecific competition (red), intraspecific competition (green), and no competition (blue). Pearson correlation coefficients (*r*) and associated *p*-values are shown in each panel to indicate the strength and significance of the relationships. The fitted regression lines and confidence intervals illustrate the direction and variation of SRL–biomass relationships across competition treatments.

**Figure S6.** Correlation plots showing pairwise relationships between above-ground resource acquisition traits and biomass accumulation under different competition treatments. Panels represent correlations between (a) specific stem length (SSL) and below-ground biomass, (b) specific stem length (SSL) and above-ground biomass, (c) specific stem length (SSL) and total biomass, (d) specific leaf area (SLA) and below-ground biomass, (e) specific leaf area (SLA) and above-ground biomass, and (f) specific leaf area (SLA) and total biomass. Each point represents an individual pot, with competition treatments indicated by color: interspecific competition (red), intraspecific competition (green), and no competition (blue). Pearson correlation coefficients (*r*) and associated *p*-values are shown in each panel to indicate the strength and significance of the relationships. The fitted regression lines and confidence intervals illustrate the direction and variation of relationships between functional traits and biomass components across competition treatments.
